# Supplementary figures and images for: Novel binding partners of the Vacuolar Transporter Chaperone (VTC) complex in Acidocalcisomes of Leishmania tarentolae
Source: PLoS Negl Trop Dis. 2026 Jul 13;20(7):e0014511. doi: 10.1371/journal.pntd.0014511 (PMC13375126; doi:10.1371/journal.pntd.0014511)

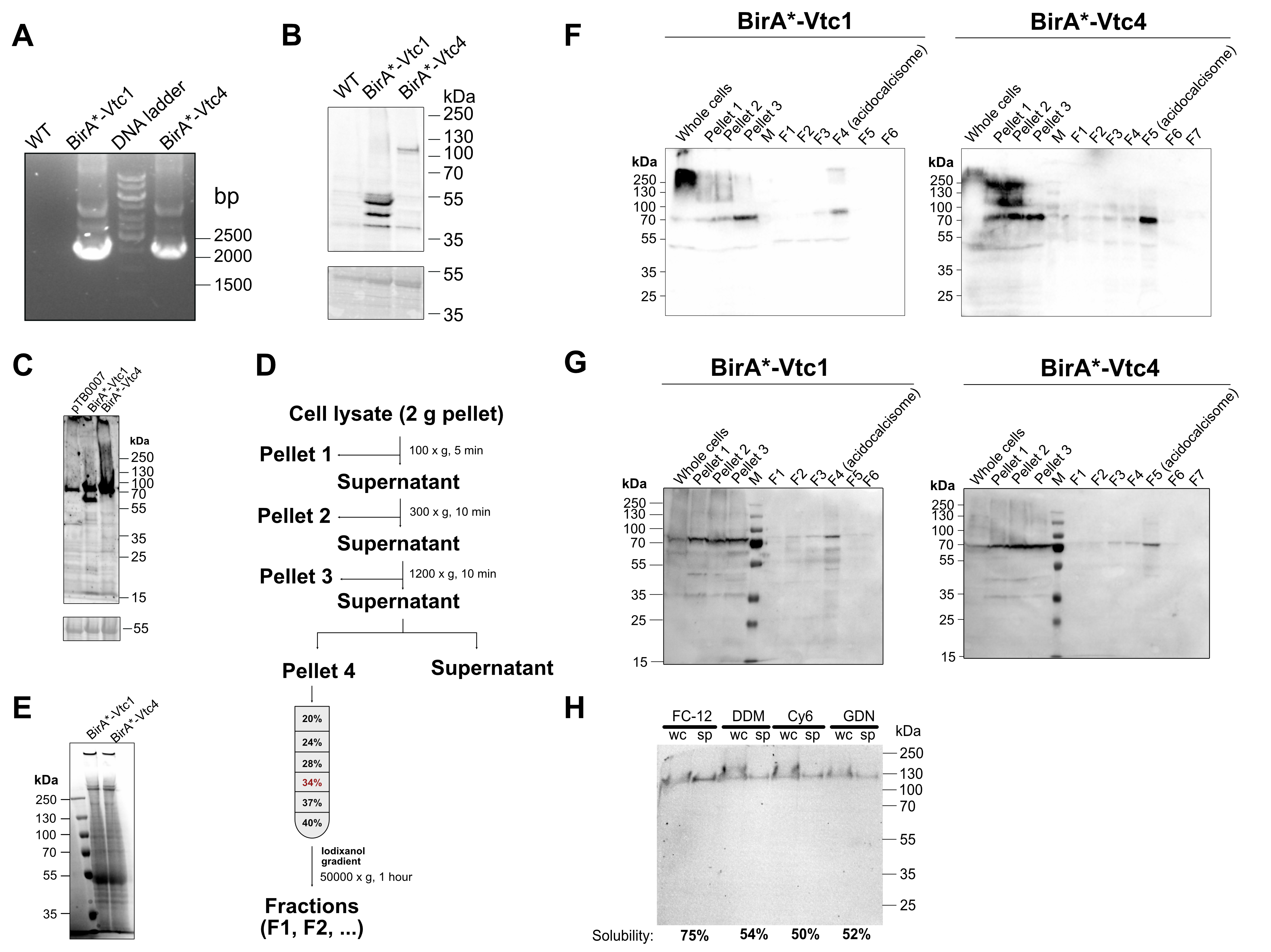

Supplement: S1 Fig — (A) PCR product of BirA*-tagging cassette integration in LtVtc1 and LtVtc4 shows clear bands around 2200 bp for both, while no bands are seen in the wt lane. (B) Western blot detection of BirA*-tagged LtVtc1/LtVtc4 expressed in L. tarentolae using anti-Myc. Clear bands are observed in LtVtc1 and LtVtc4 lanes and absent in WT. Ponceau S staining of the membrane is shown below as loading control. (C) Western blot of cells expressing BirA* tagged LtVtc1 and Vtc4 after being treated with 50 µM biotin for 40h. pTB007: cells containing the pTB007 plasmid as a negative control. Ponceau S staining of the membrane is shown below as a loading control. (D) Acidocalcisome fractionation protocol. (E) Acidocalcisome fraction after iodixanol gradient ultracentrifugation. (F) Western blot of the iodixanol gradient fractionation using an anti-TmPPase antibody. (G) Western blot of the iodixanol gradient fractionation using Avidin-HRP. (H) SDS-PAGE showing the mNG fluorescence signal from the detergent solubilisation assay of mNG-LtVtc4 using FC-12 (Fos-choline-12), DDM (n-dodecyl-β-maltoside), Cy6 (Cymal-6), and GDN (Glyco-Diosgenin). wc = whole cell sample, sp = supernatant after centrifugation. (PNG) [file pntd.0014511.s001.png]

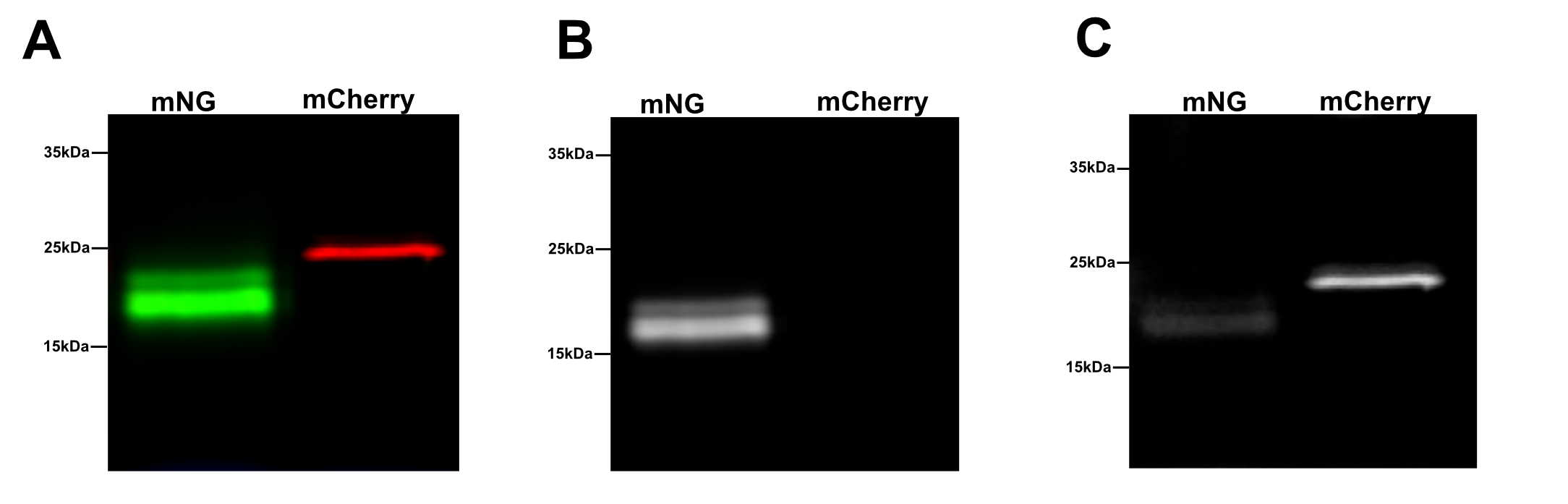

Supplement: S2 Fig — (A) Merged visualisation of purified mNG and mCh tags. (B) Signal of the purified mNG tag alone, displaying exclusively green fluorescence in the 488 nm channel. (C) Signal of the purified mCh tag alone in the 532 nm channel, showing strong red fluorescence and weaker green fluorescence due to spectral bleed-through from weak excitation at 532 nm and the emission tail into the red detection range. As a result, merged images may display yellowish bands, depending on the relative green signal intensity. (PNG) [file pntd.0014511.s002.png]

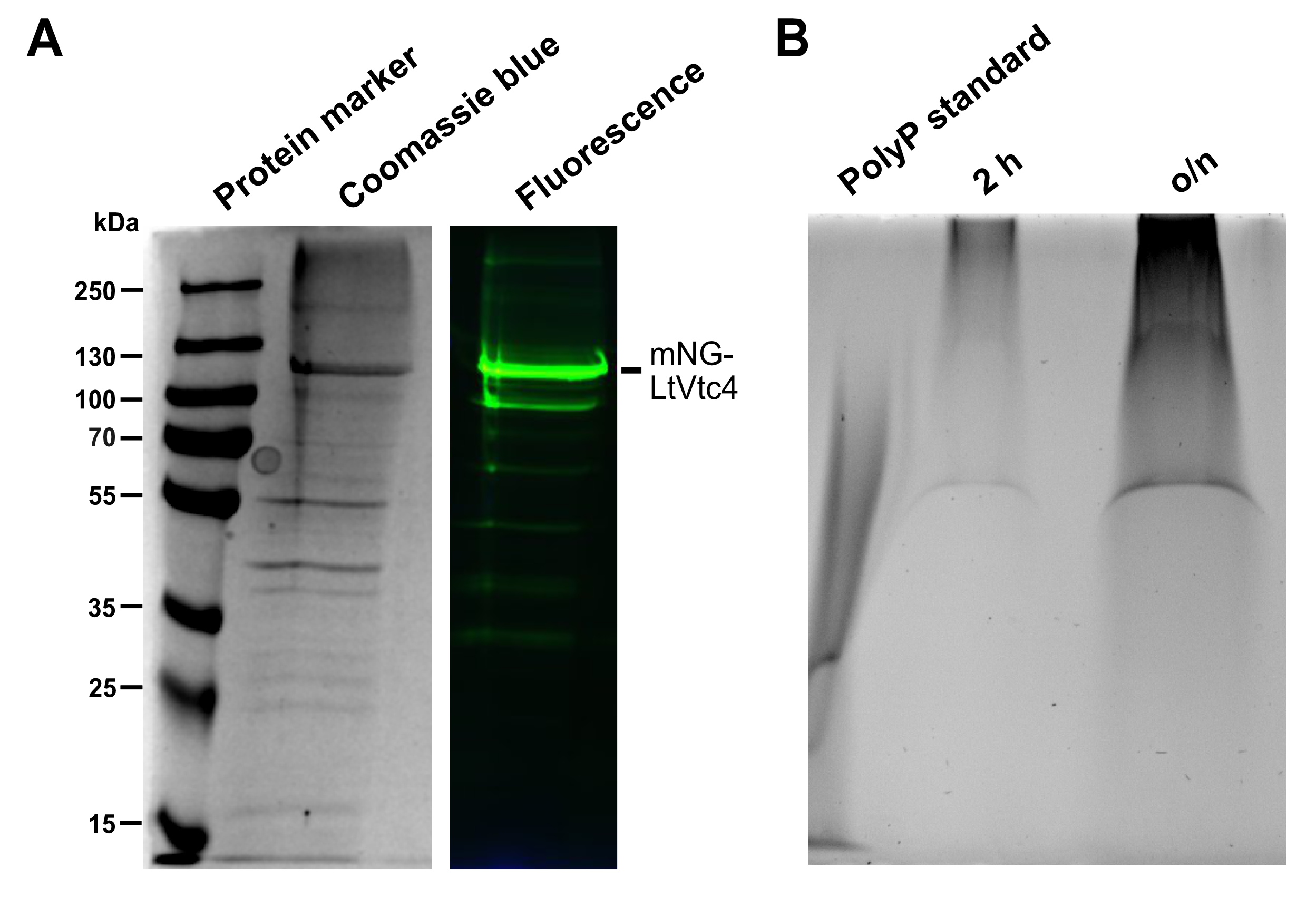

Supplement: S3 Fig — (A) SDS–PAGE analysis of the purified VTC complex visualised by Coomassie Brilliant Blue staining and in-gel fluorescence showing mNG-LtVtc4 signal. (B) Urea–PAGE analysis of polyP synthesis activity of the purified VTC complex. The image shows 0.1 µg of polyP standard and polyP produced by the purified VTC complex after incubation with ATP and IP6 at 30 °C for 2 h and overnight. (PNG) [file pntd.0014511.s003.png]

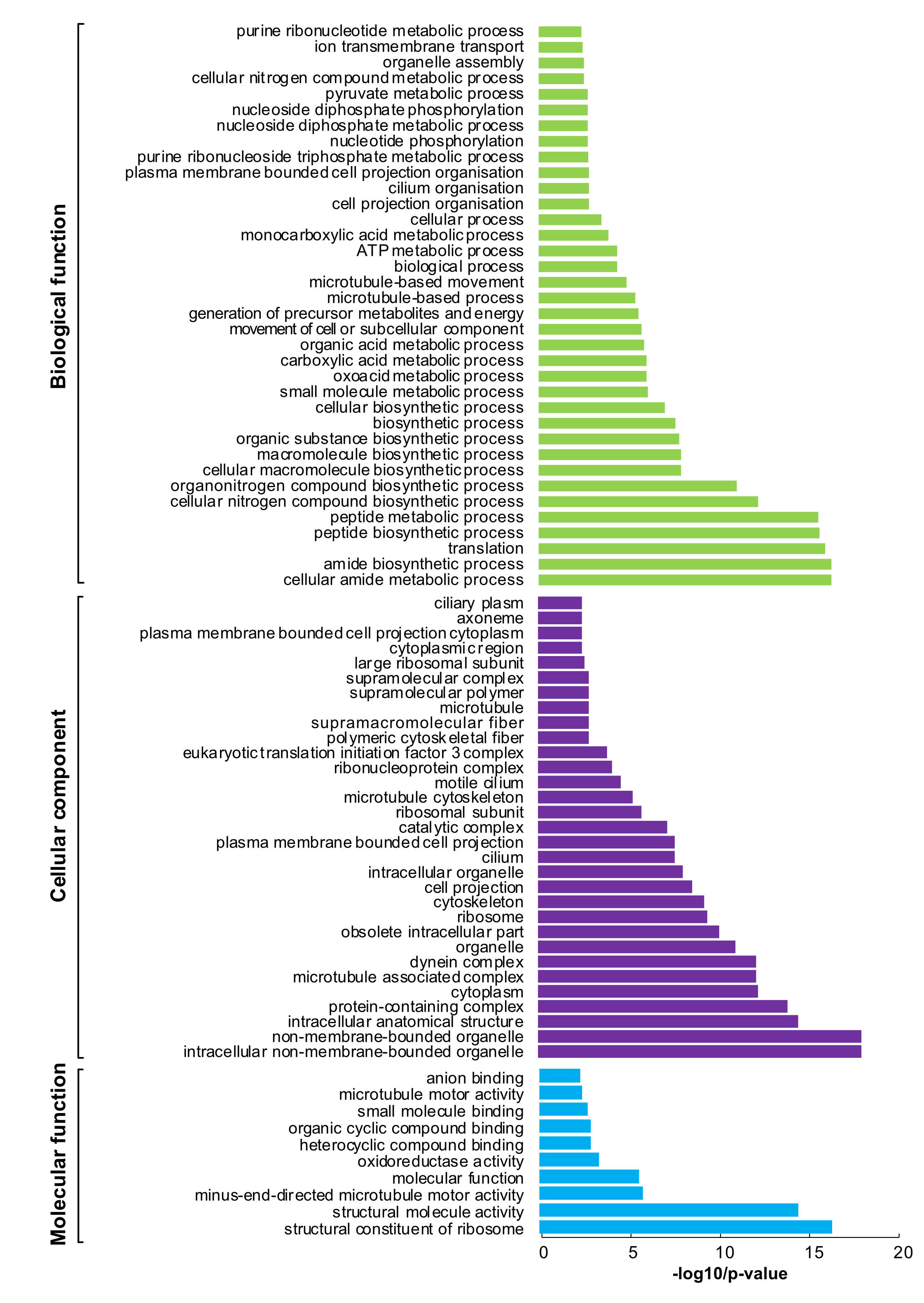

Supplement: S4 Fig — (PNG) [file pntd.0014511.s004.png]

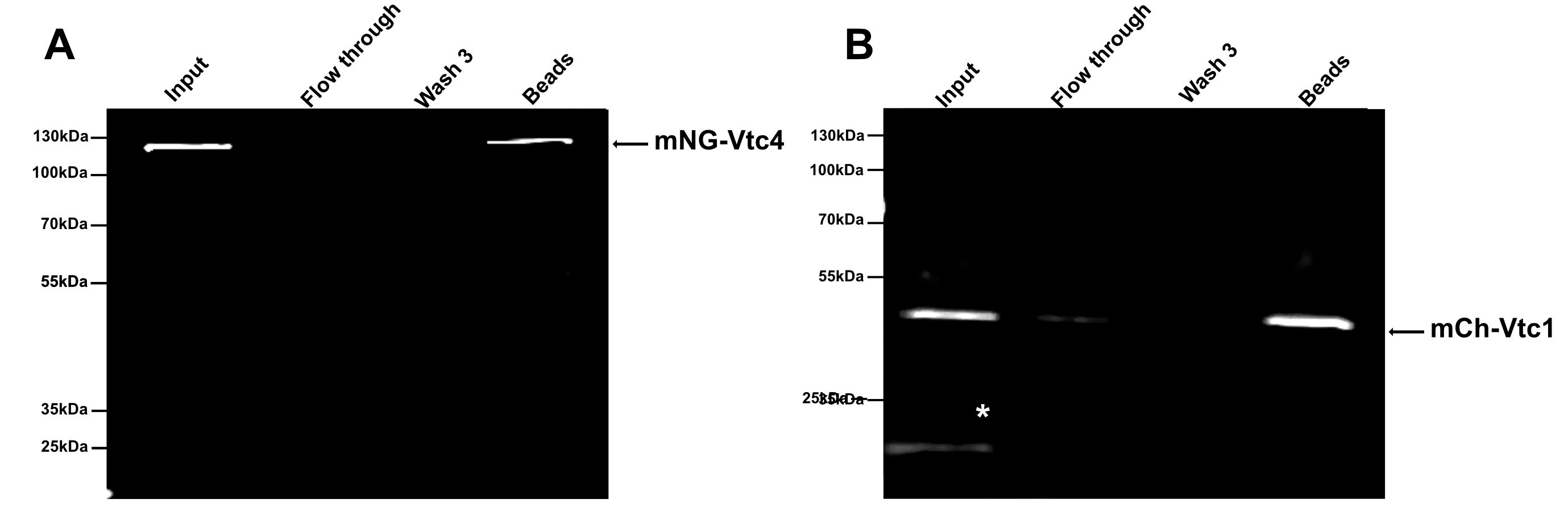

Supplement: S5 Fig — (A) Detection of mNG-LtVtc4 signal in the green fluorescence channel (488 nm), indicating the presence of LtVtc4 in the assay. (B) Detection of mCh-LtVtc1 signal in the red fluorescence channel (532 nm). The mNG signal may also be partially visible in this channel, depending on its relative intensity, which results in yellow bands reflecting overlapping fluorescence in the merged images. The fluorescence signal marked by a white asterisk in the gel corresponds to the mCh tag alone, visible only in the input due to partial degradation. (PNG) [file pntd.0014511.s005.png]

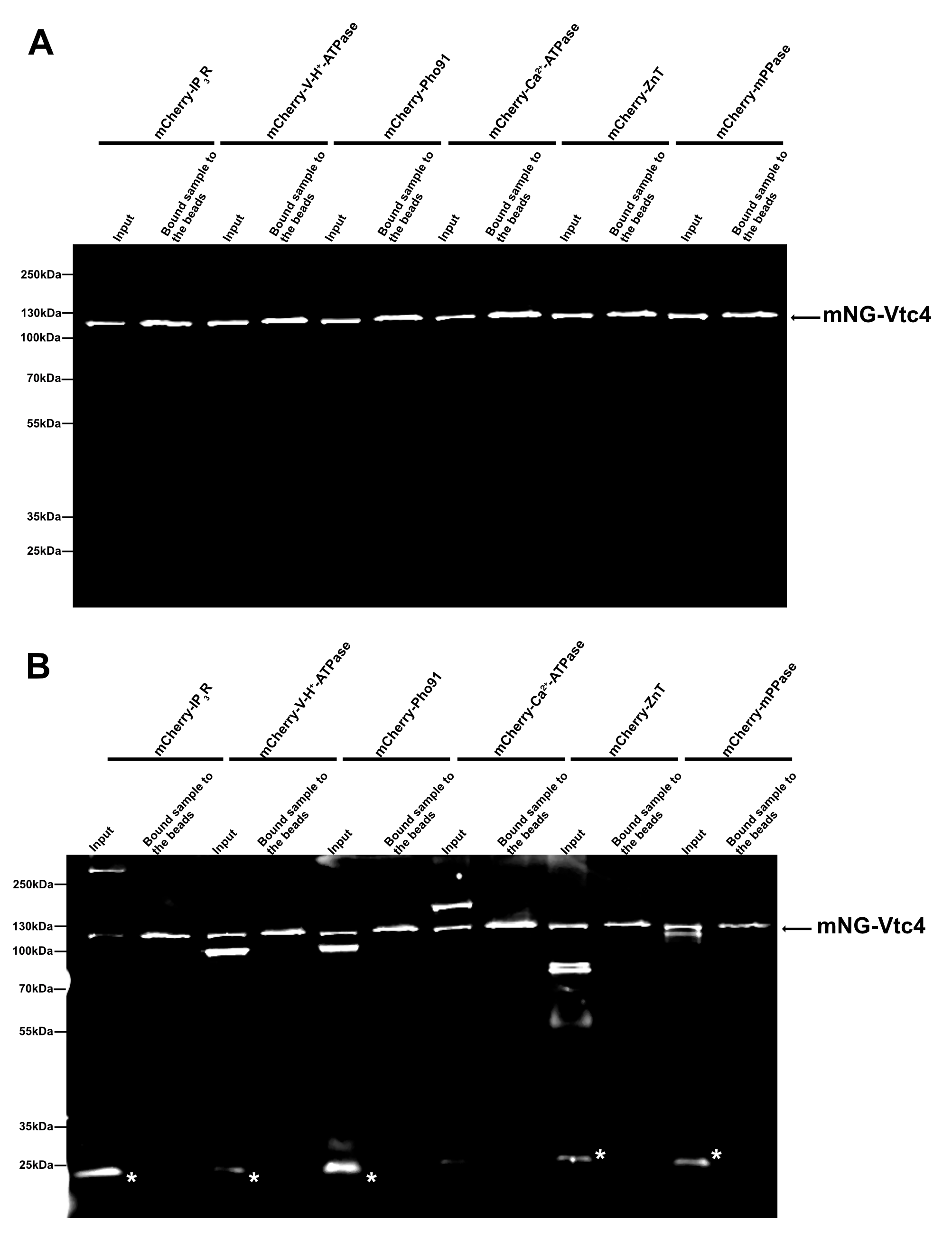

Supplement: S6 Fig — (A) Detection of mNG-LtVtc4 signal in the green fluorescence channel (488 nm), indicating the presence of Vtc4 in the assay. (B) Detection of mCh-tagged acidocalcisomal proteins in the red fluorescence channel (532 nm). The mNG signal may also be partially visible in this channel, depending on its relative intensity, which results in yellow bands reflecting overlapping fluorescence in the merged images. The fluorescence signal marked by a white asterisk in the gel corresponds to the mCh tag alone, visible only in the input due to partial degradation. (PNG) [file pntd.0014511.s006.png]

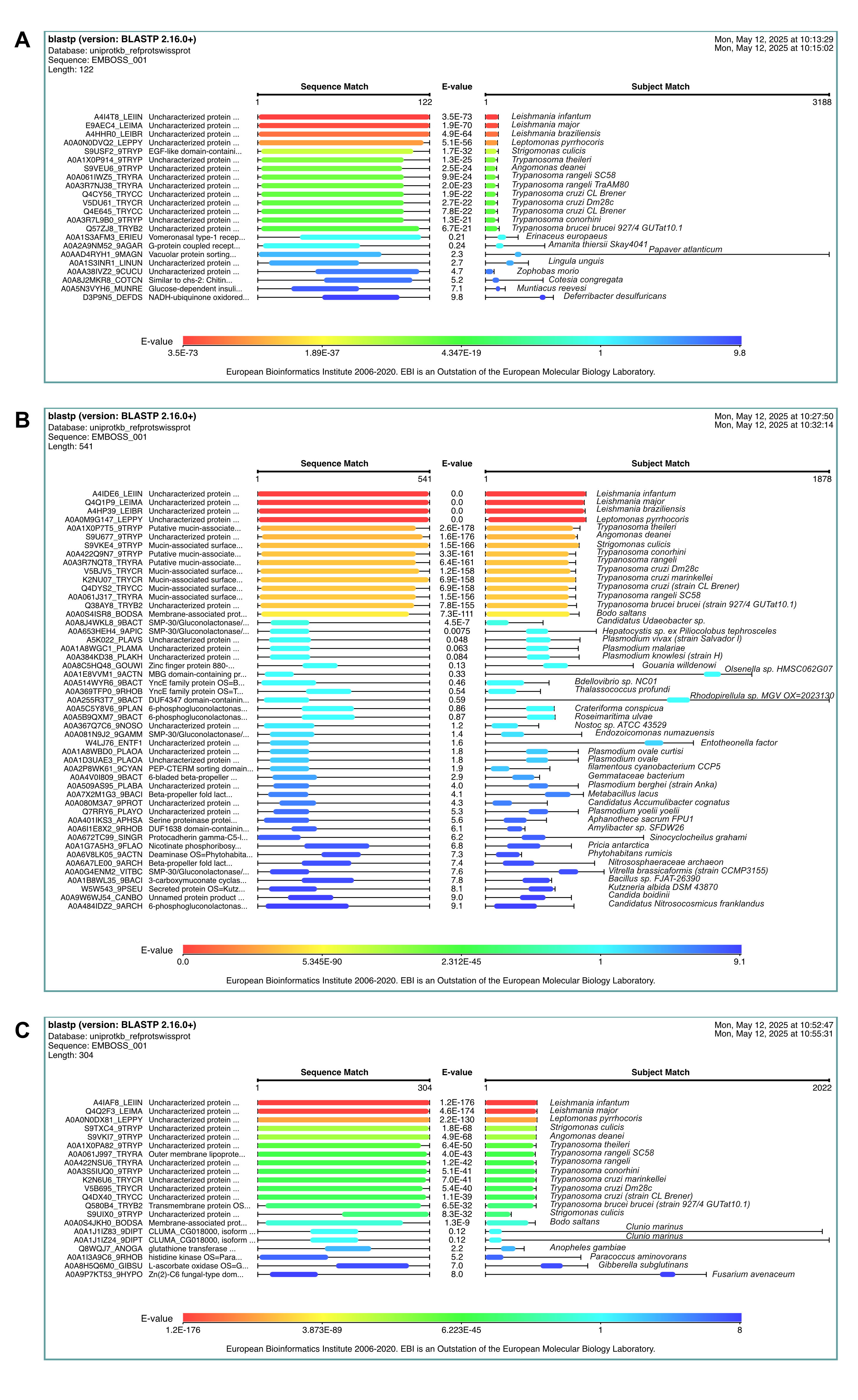

Supplement: S7 Fig — (PNG) [file pntd.0014511.s007.png]

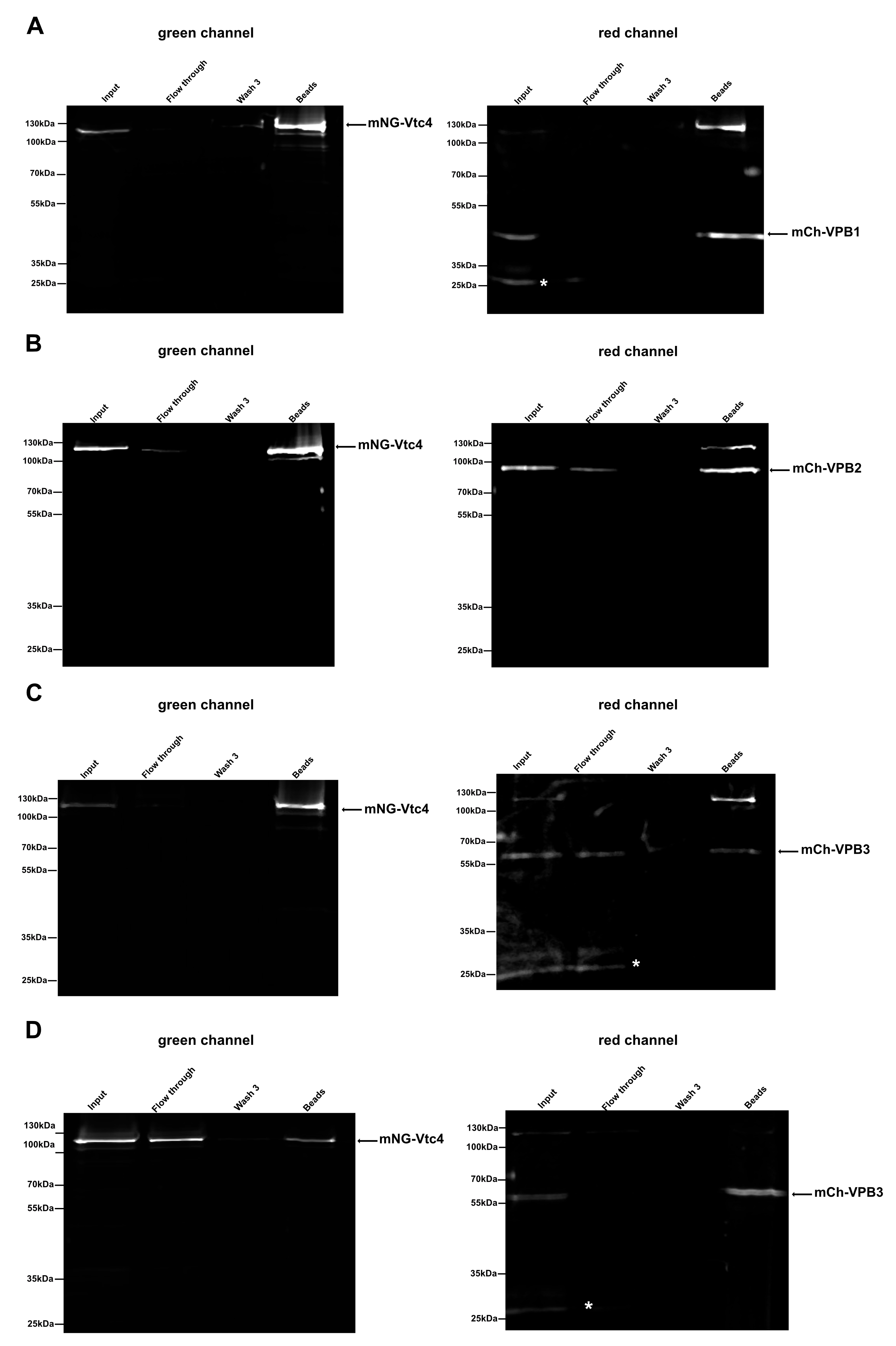

Supplement: S8 Fig — (A) Detection of mNG-LtVtc4 signal in the green fluorescence channel (488 nm), indicating the presence of Vtc4 in the assay and detection of LtVBP1-mCh in the red fluorescence channel (532 nm) using mNG Trap Agarose Beads. (B) Detection of mNG-LtVtc4 signal in the green fluorescence channel (488 nm), indicating the presence of Vtc4 in the assay and detection of LtVBP2-mCh in the red fluorescence channel (532 nm) using mNG Trap Agarose Beads. (C) Detection of mNG-LtVtc4 signal in the green fluorescence channel (488 nm), indicating the presence of Vtc4 in the assay and detection of LtVBP3-mCh in the red fluorescence channel (532 nm) using mNG Trap Agarose Beads. (D) Detection of mNG-LtVtc4 signal in the green fluorescence channel (488 nm), indicating the presence of Vtc4 in the assay and detection of LtVBP3-mCh in the red fluorescence channel (532 nm) using RFP-Trap Magnetic Particles M270. The mNG signal may also be partially visible in red channel, depending on its relative intensity, which results in yellow bands reflecting overlapping fluorescence in the merged images. The fluorescence signal marked by a white asterisk in the gel corresponds to the mCh tag alone, visible only in the input due to partial degradation. (PNG) [file pntd.0014511.s008.png]

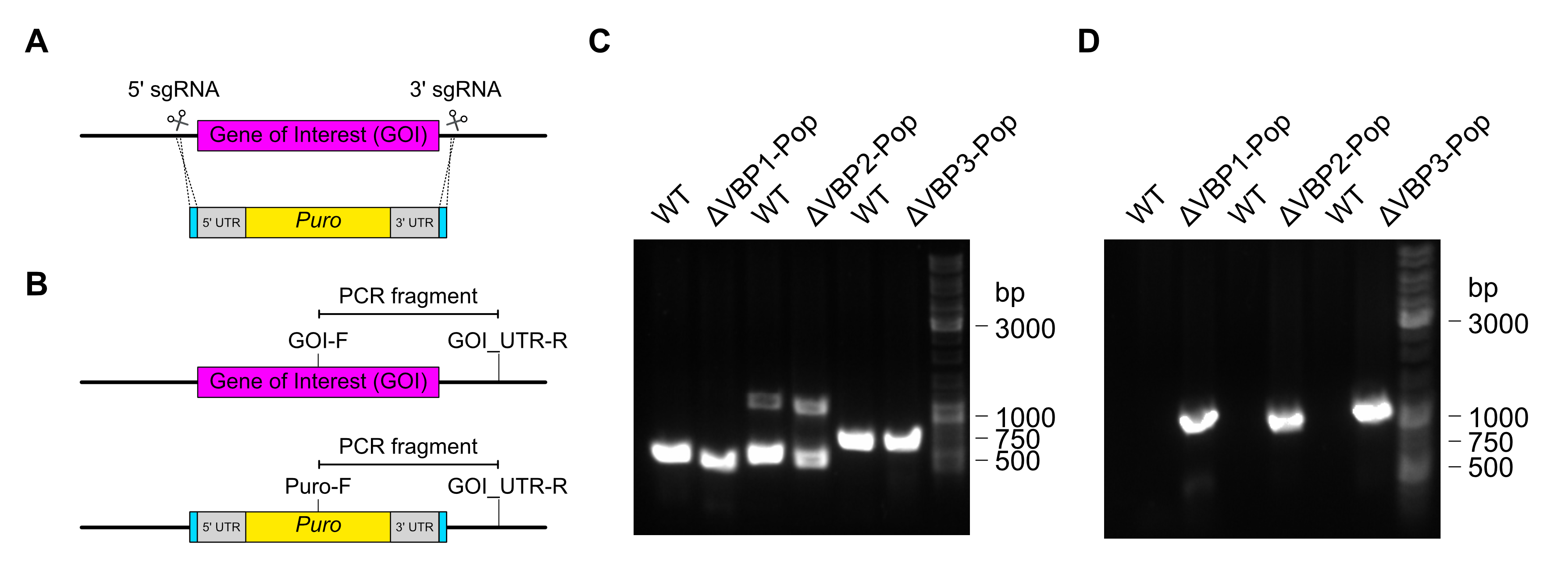

Supplement: S9 Fig — (A) Schematic representation of the strategy used to knock out the VBP genes. Two sgRNAs, targeting the 5′ and 3′ ends of the coding sequence (CDS) of the gene of interest (GOI; scissors), were used to direct Cas9-mediated removal of the complete VBP CDS. A donor DNA containing a puromycin resistance marker (yellow) flanked by 30-nt homology arms (cyan) was also delivered to mediate double-strand break repair. (B) Schematic representation of the two PCR assays used to screen for the presence or absence of the GOI and the puromycin resistance marker in the parental line and transfectant populations (ΔVBP1-pop, ΔVBP2-pop, and ΔVBP3-pop). (C) PCR amplification of fragments from the parental line and knockout transfectant populations using a GOI-specific forward primer (VBP1-F, VBP2-F, or VBP3-F) together with the corresponding 3′-UTR reverse primer (VBP1_UTR-R, VBP2_UTR-R, or VBP3_UTR-R). These primer pairs amplify fragments of 563, 568, and 739 bp, respectively. In all cases, both the parental line and knockout transfectant populations retained the gene. (D) PCR amplification of fragments from the parental line and knockout transfectant populations using the puromycin resistance marker forward primer (Puro-F) together with the corresponding 3′-UTR reverse primer (VBP1_UTR-R, VBP2_UTR-R, or VBP3_UTR-R). These primer pairs amplify fragments of 1204, 1200, and 1191 bp, respectively. In all cases, only the knockout transfectant populations produced the corresponding PCR fragments, whereas the parental line did not. Overall, the PCR amplification results shown in C and D indicate that the knockout transfectant populations contain the puromycin resistance cassette while still retaining the parental VBP gene signal. This suggests that these populations may contain cells with disrupted VBP loci together with cells or alleles retaining the parental VBP genes. (PNG) [file pntd.0014511.s009.png]

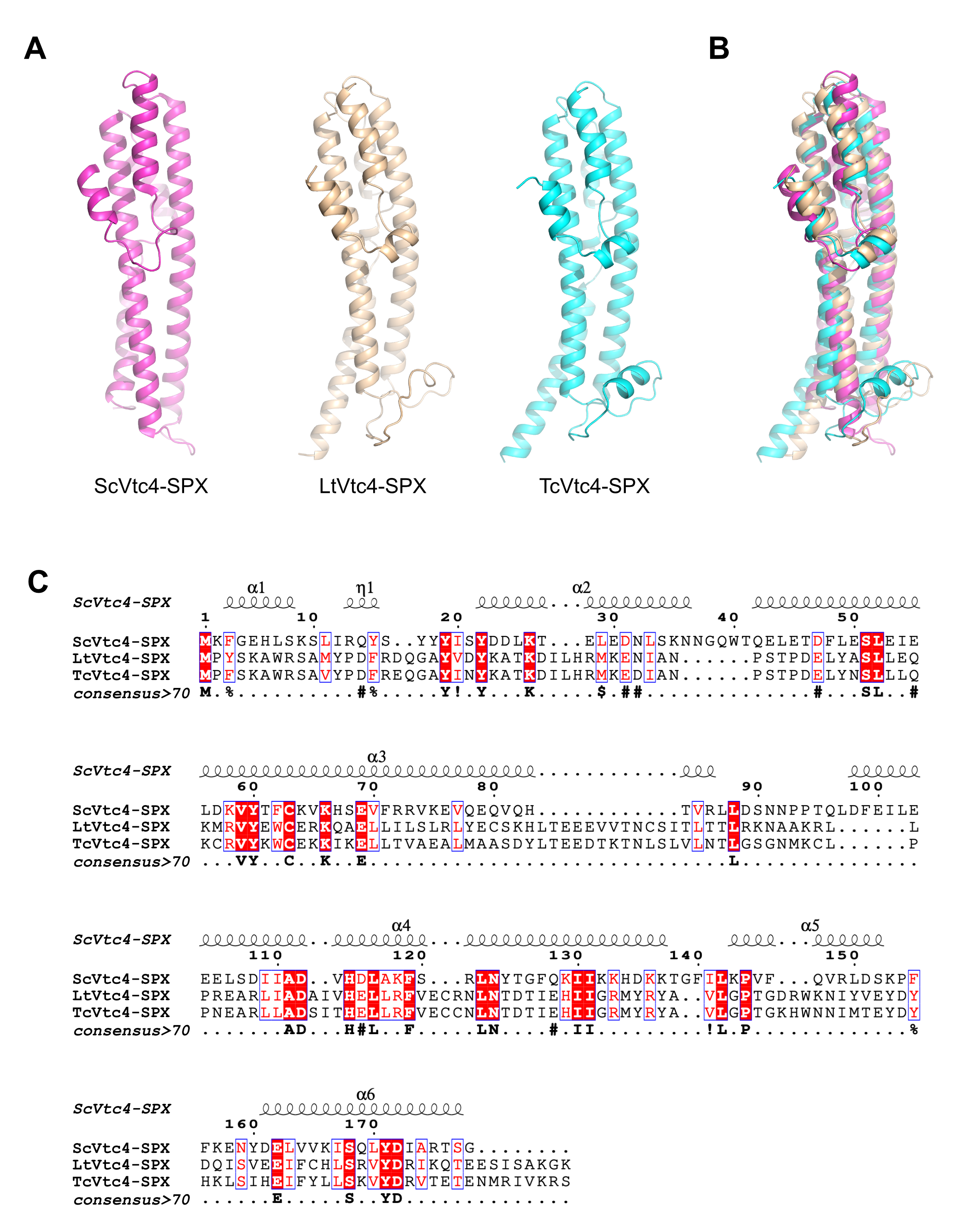

Supplement: S10 Fig — (A) Structure of yeast ScVtc4-SPX, generated from PDB ID 8I6V (magenta), and AlphaFold3 models of LtVtc4-SPX (wheat) and Trypanosoma cruzi Vtc4-SPX (cyan). All three structures adopt a typical SPX domain fold. (B) Structure superposition of the three structures shown in A. (C) Sequence alignment of ScVtc4-SPX, LtVtc4-SPX, and TcVtc4-SPX. The red box shows sequence identity, and the blue rectangle shows consensus > 70%. The secondary structure corresponds to the solved structure of yeast ScVtc4-SPX, generated from PDB ID 8I6V (15). (PNG) [file pntd.0014511.s010.png]

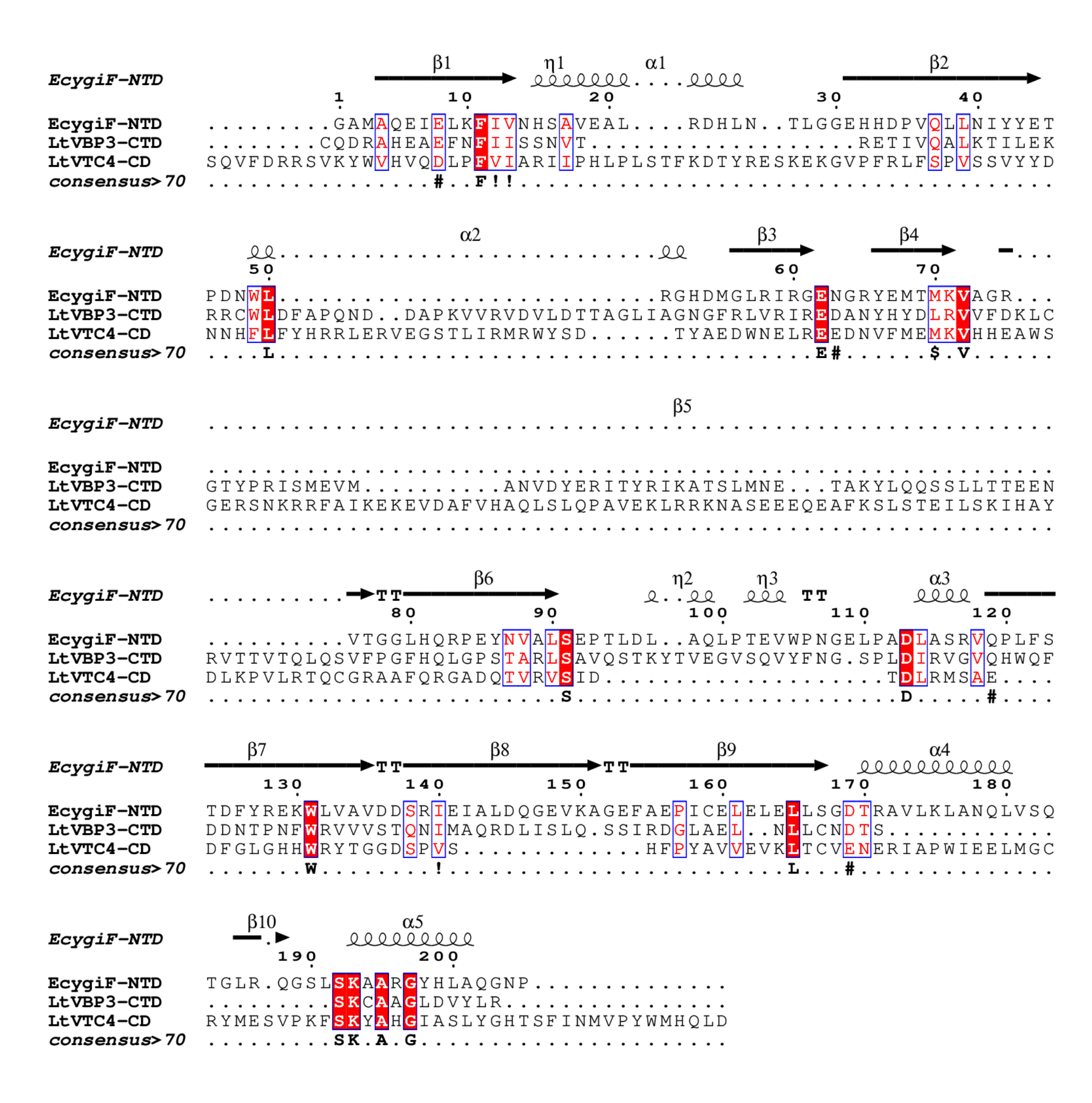

Supplement: S11 Fig — The red box shows sequence identity, and the blue rectangle shows consensus > 70%. The secondary structure corresponds to the solved structure of EcygiF (54). (PNG) [file pntd.0014511.s011.png]

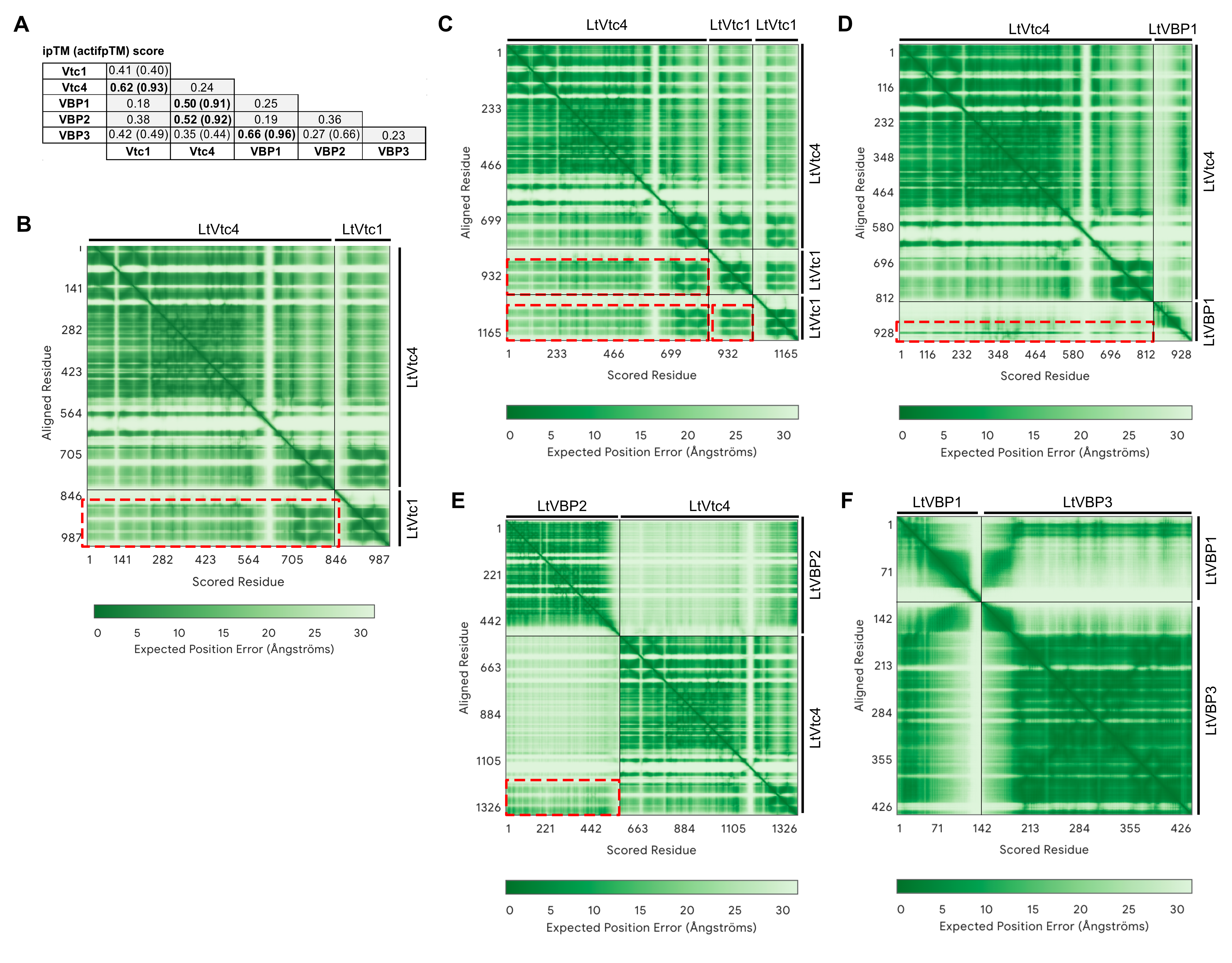

Supplement: S12 Fig — (A) ipTM and actifpTM scores for pairwise interactions among LtVtc1, LtVtc4, LtVBP1, LtVBP2, and LtVBP3. (B-F) PAE plot for complex structure prediction of: (B) LtVtc1 with LtVtc4, (C) two LtVtc1 with one LtVtc4, (D) LtVBP1 with LtVtc4, (E) LtVBP2 with LtVtc4, and (F) LtVBP1 with LtVBP3. The red-dashed rectangle highlights inter-subunit regions with low alignment error, indicating high confidence in interchain interactions. (PNG) [file pntd.0014511.s012.png]
