# Supplementary material for: Novel binding partners of the Vacuolar Transporter Chaperone (VTC) complex in Acidocalcisomes of Leishmania tarentolae
Source: PLoS Negl Trop Dis. 2026 Jul 13;20(7):e0014511. doi: 10.1371/journal.pntd.0014511 (PMC13375126; doi:10.1371/journal.pntd.0014511)

Fig 1

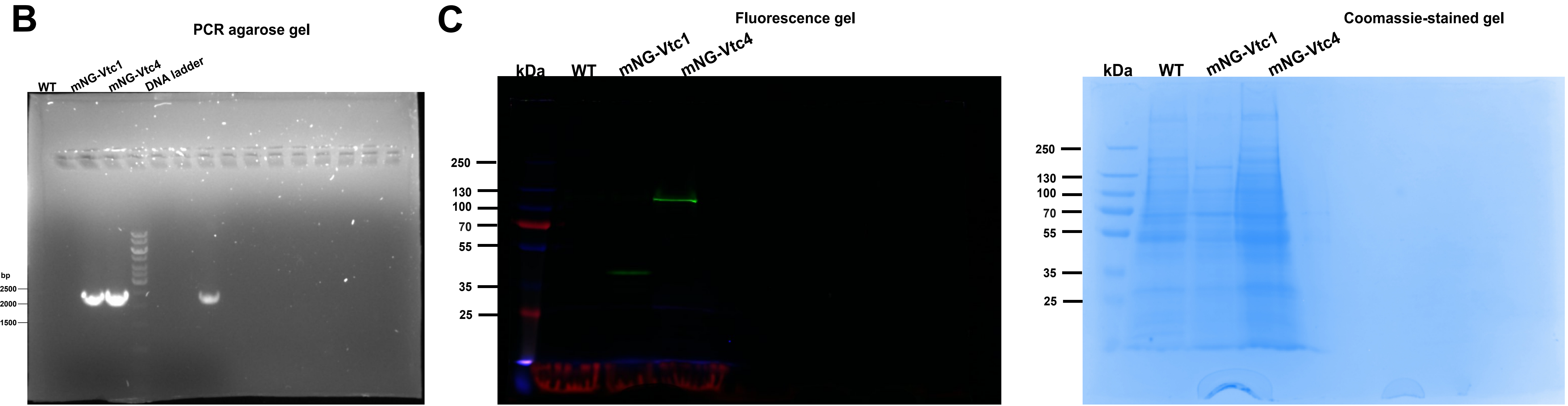
B
PCR agarose gel
WT
mNG-Vtc1
mNG-Vtc4
DNA ladder
bp
2500
2000
1500
C
Fluorescence gel
kDa
WT
mNG-Vtc1
mNG-Vtc4
250
130
100
70
55
35
25
Coomassie-stained gel
kDa
WT
mNG-Vtc1
mNG-Vtc4
250
130
100
70
55
35
25

Fig 4 Fluorescence gels

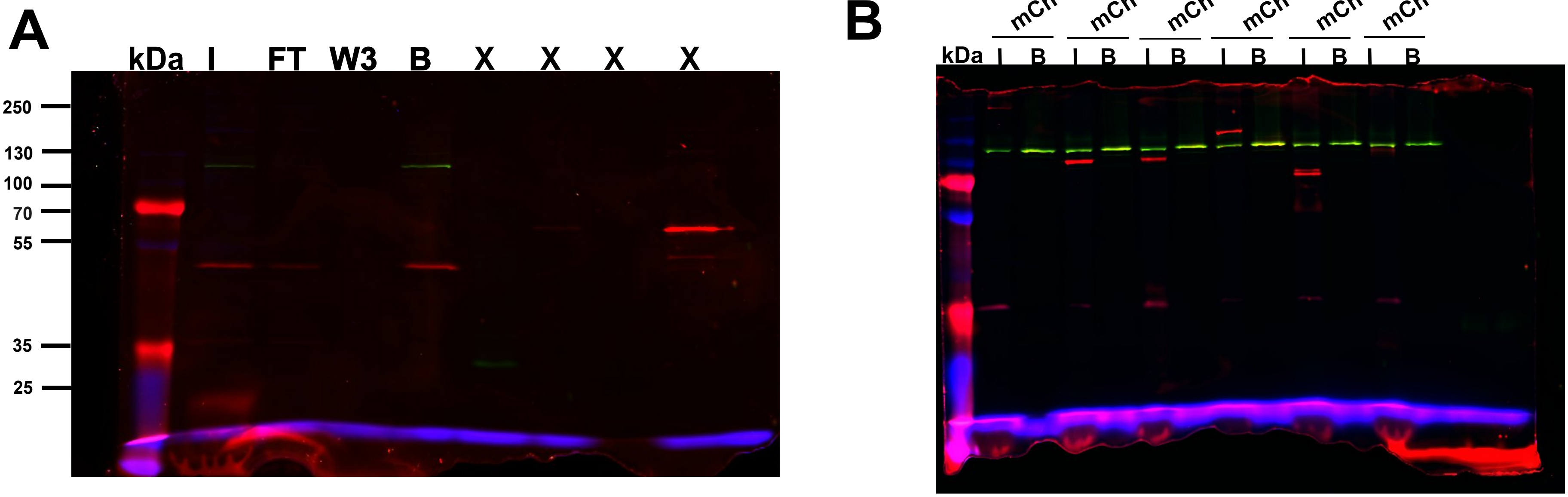

Fig 6 Fluorescence gels

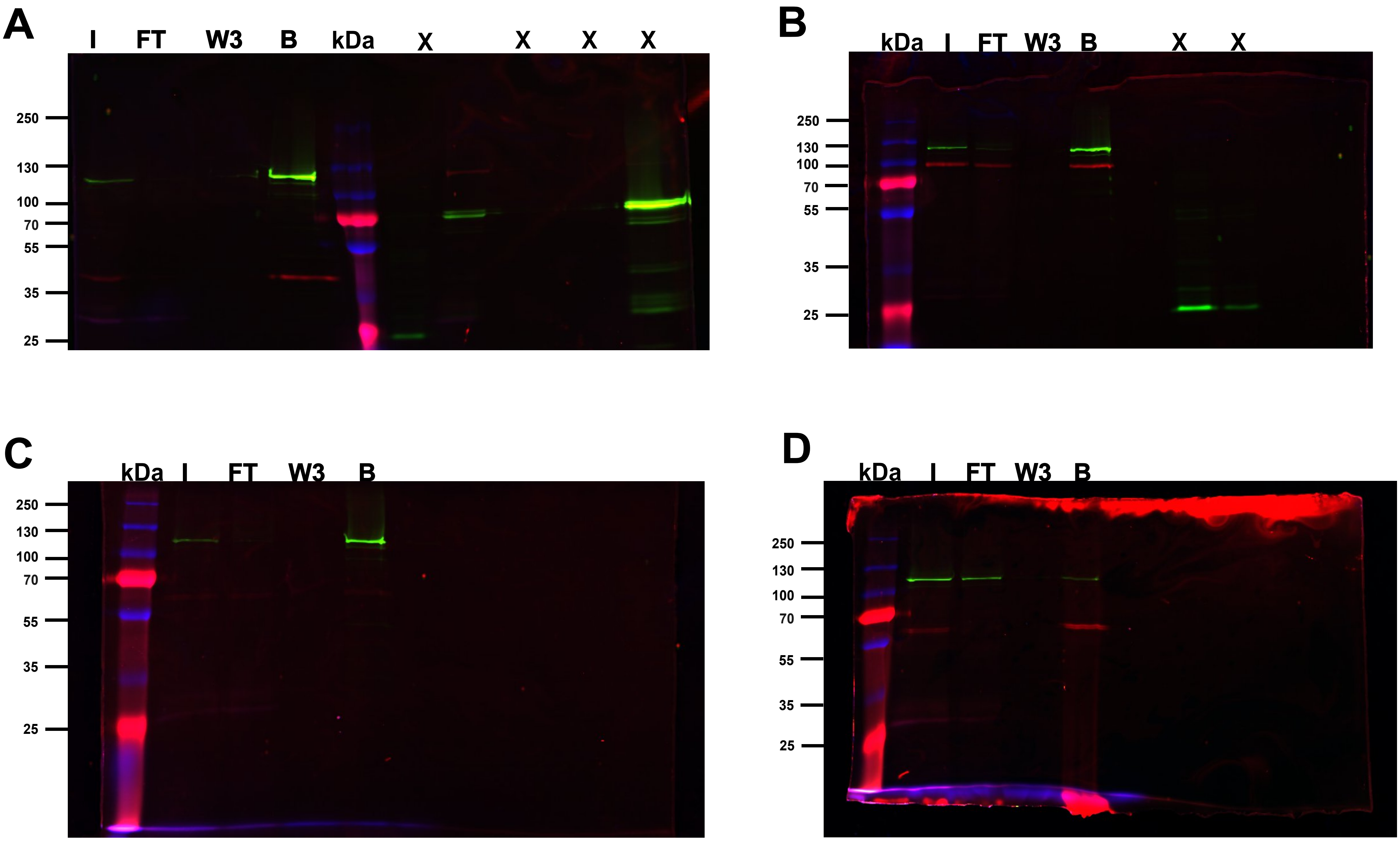

S1 Fig panel A PCR agarose gel

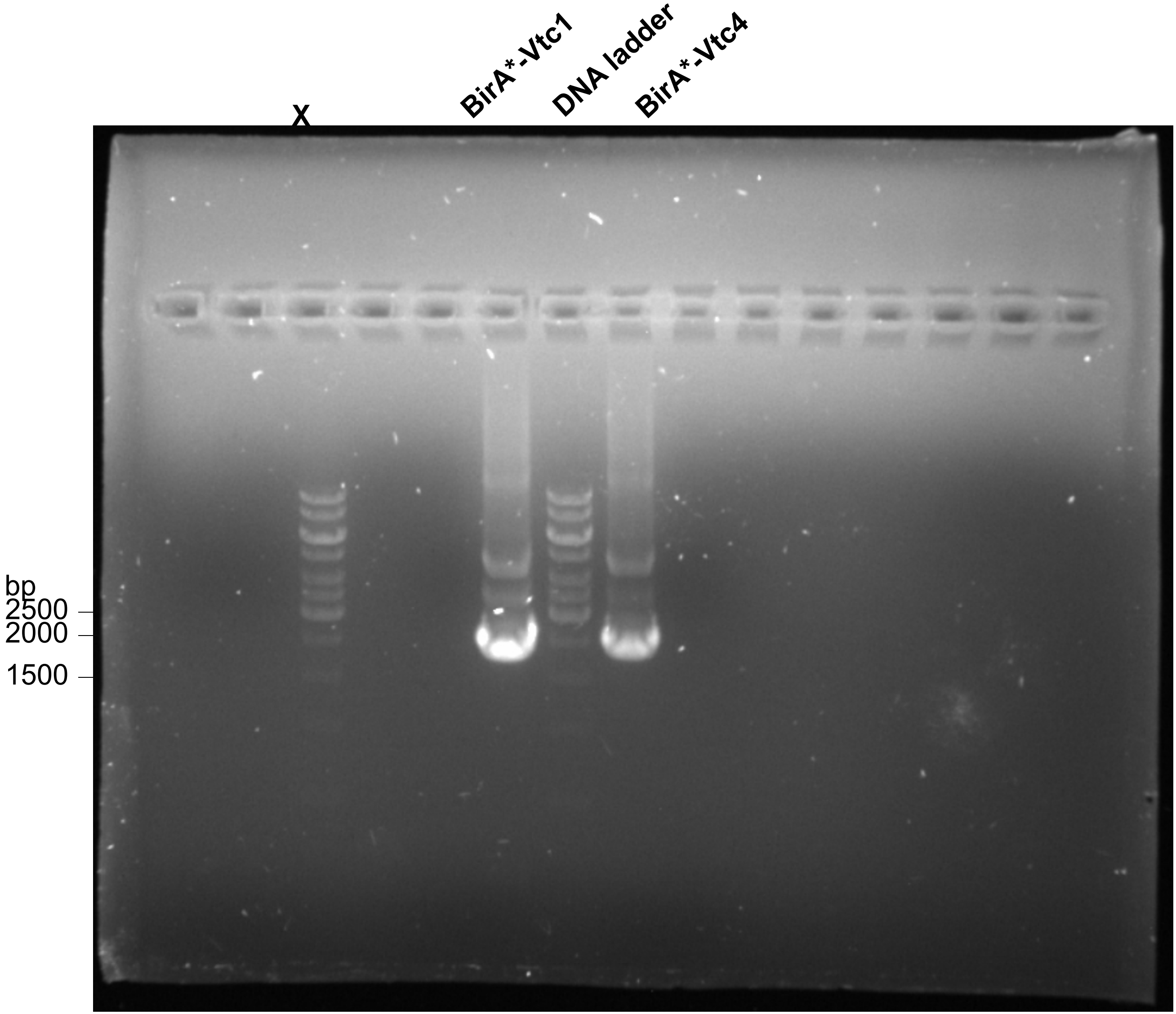

S1 Fig panel B Western blot and Ponceau S staining

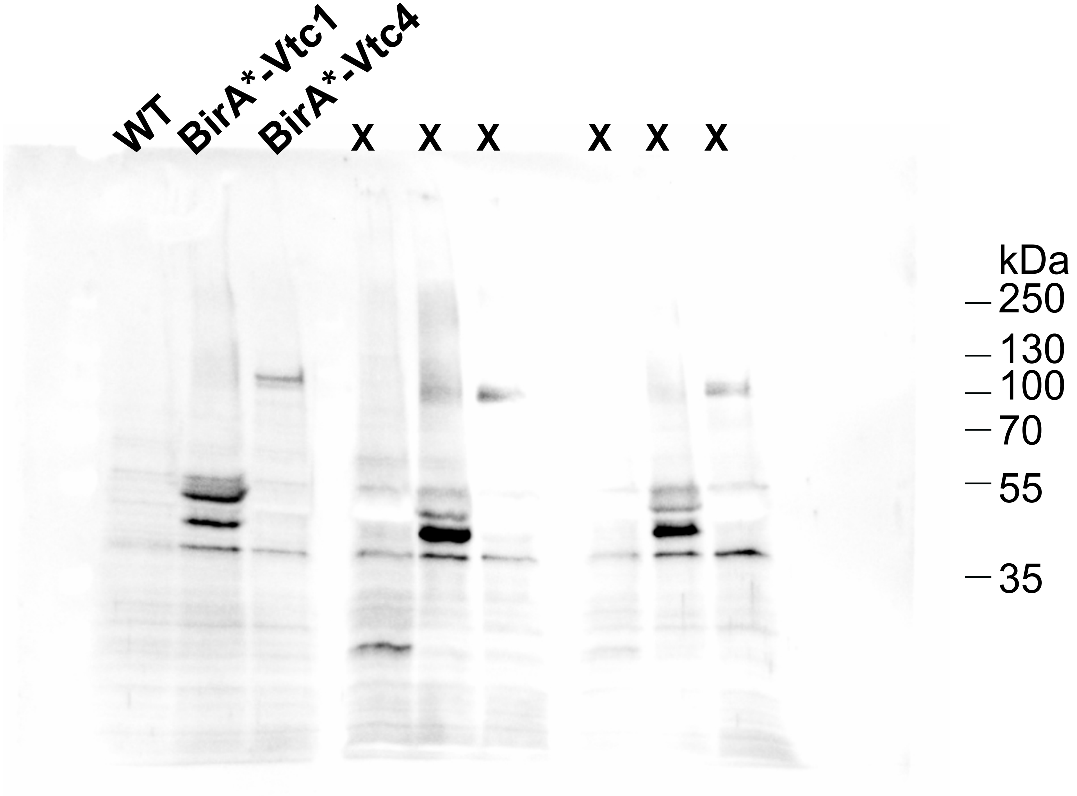

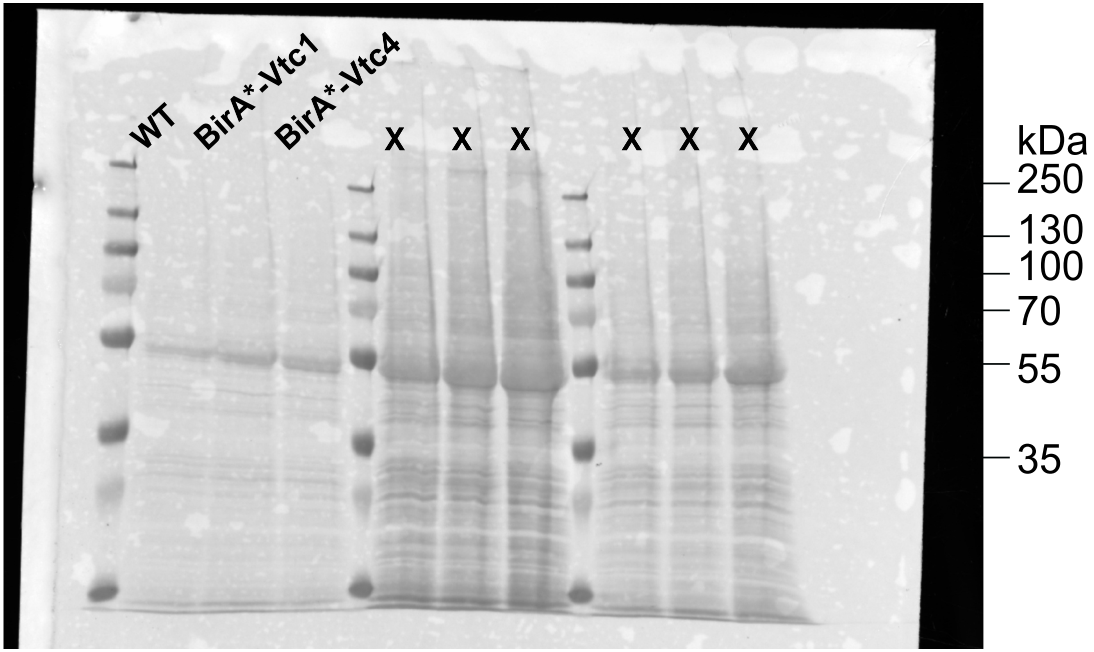

S1 Fig panel C Western blot and Ponceau S staining

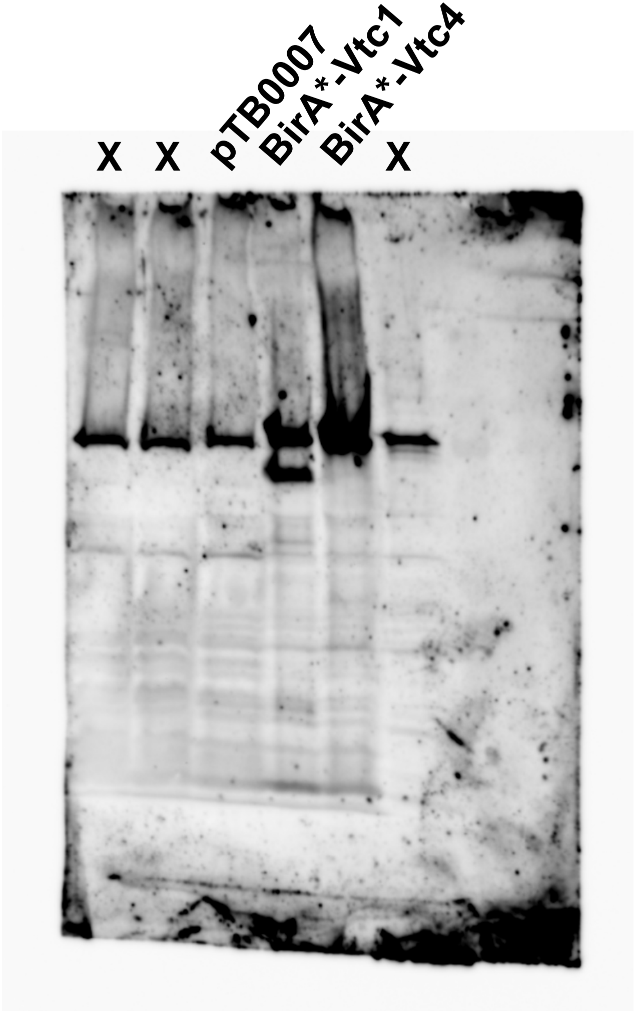

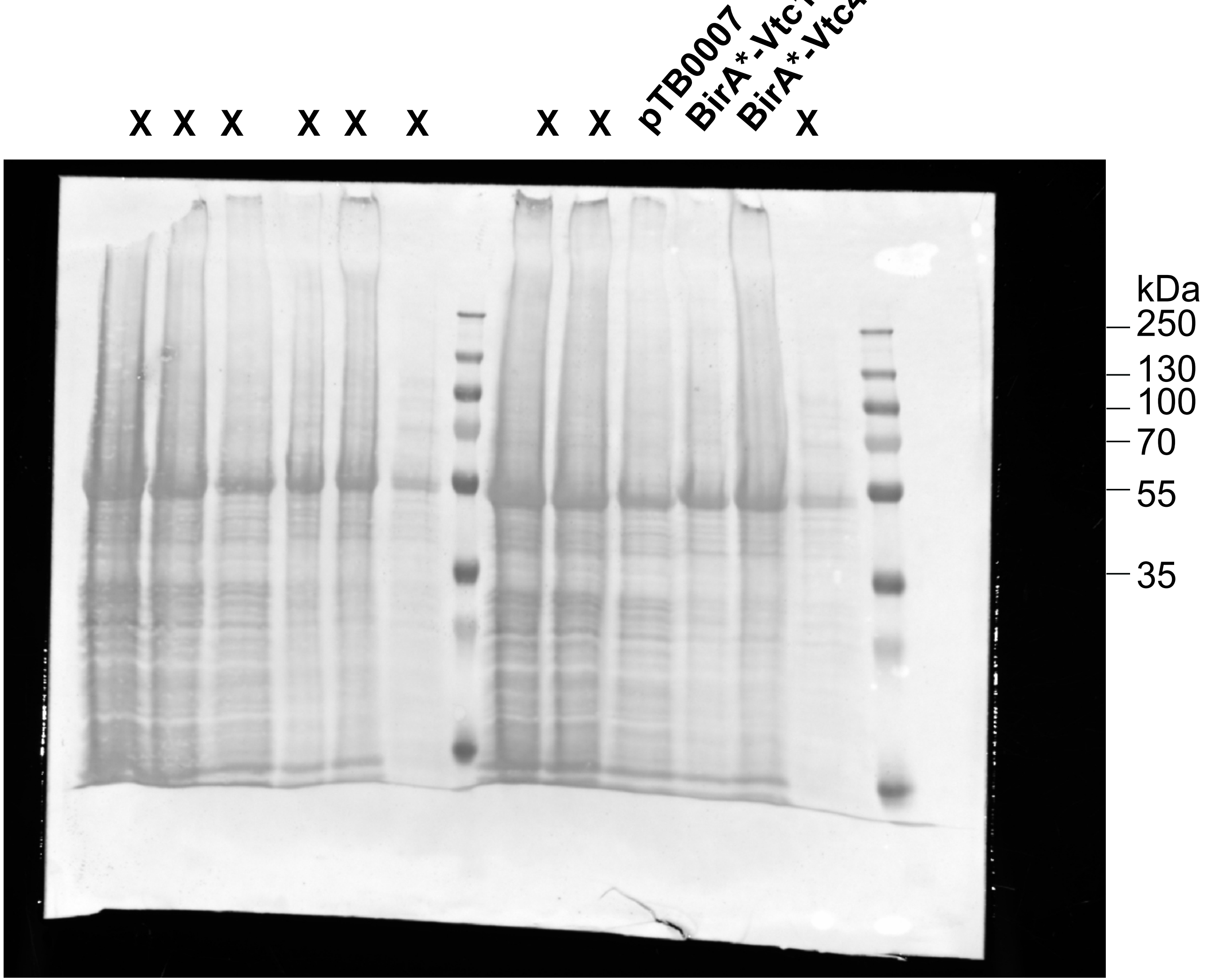

S1 Fig panel E Coomassie-stained gel

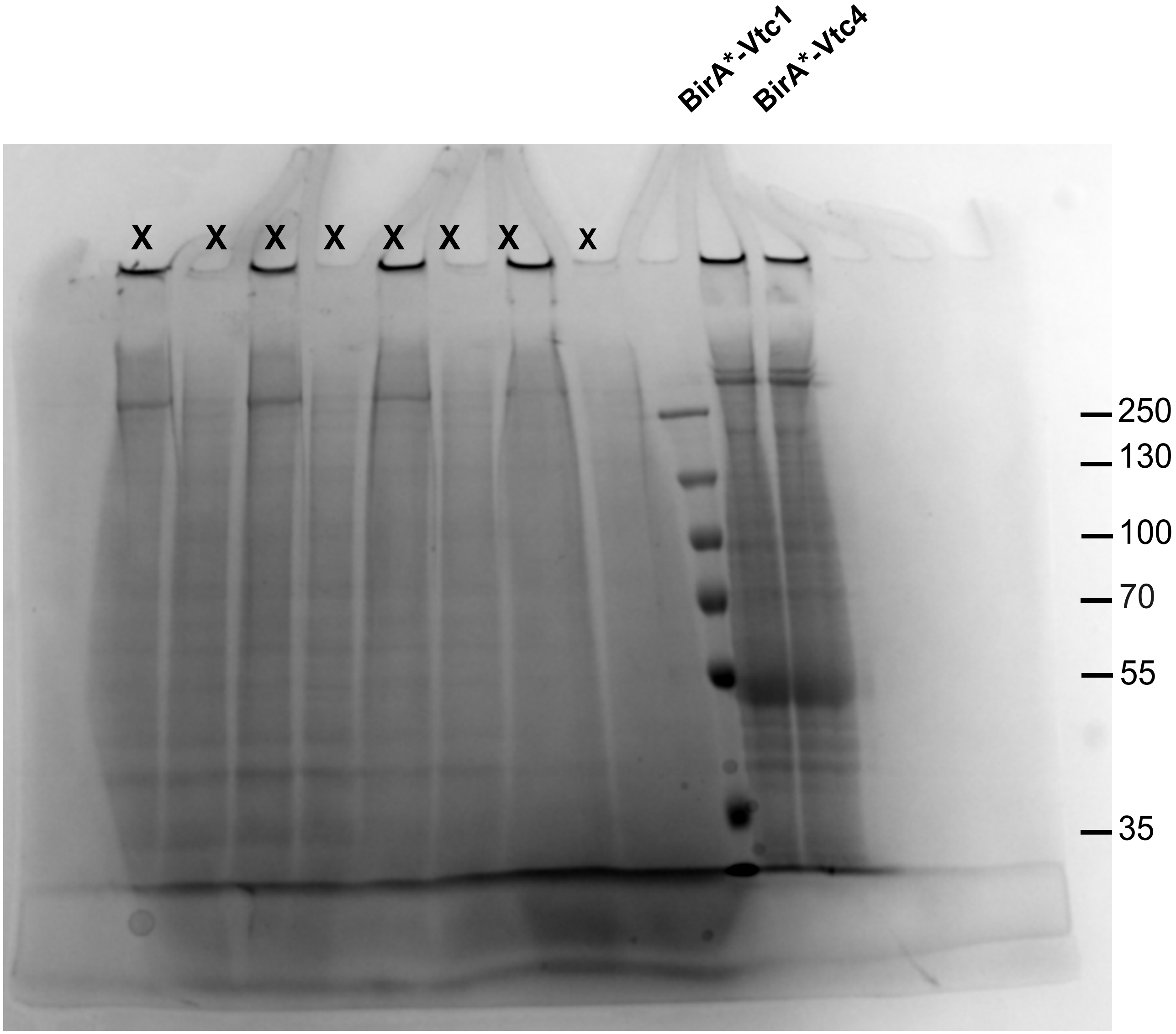

S1 Fig panel F   Western blot

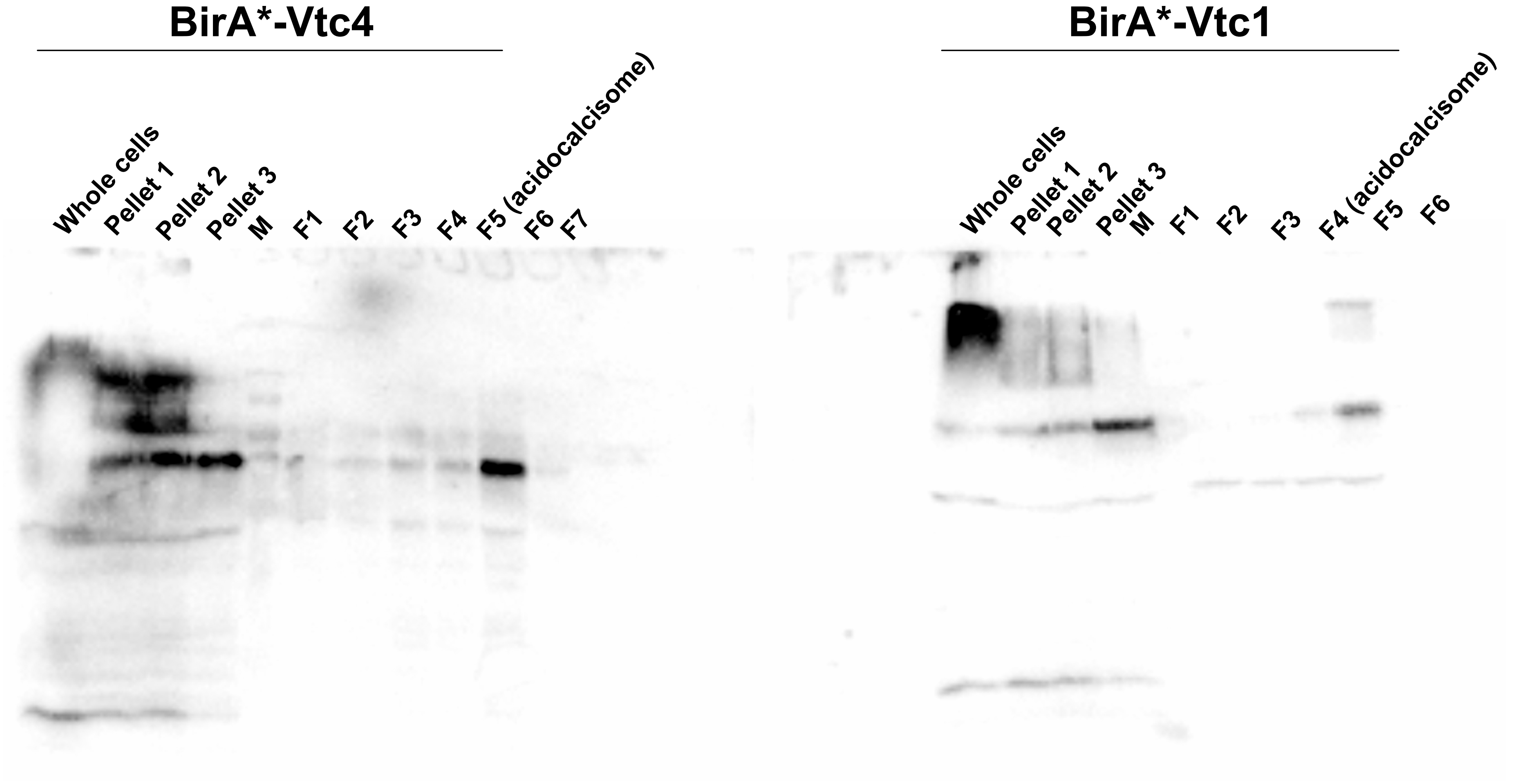

S1 Fig panel G Western blot

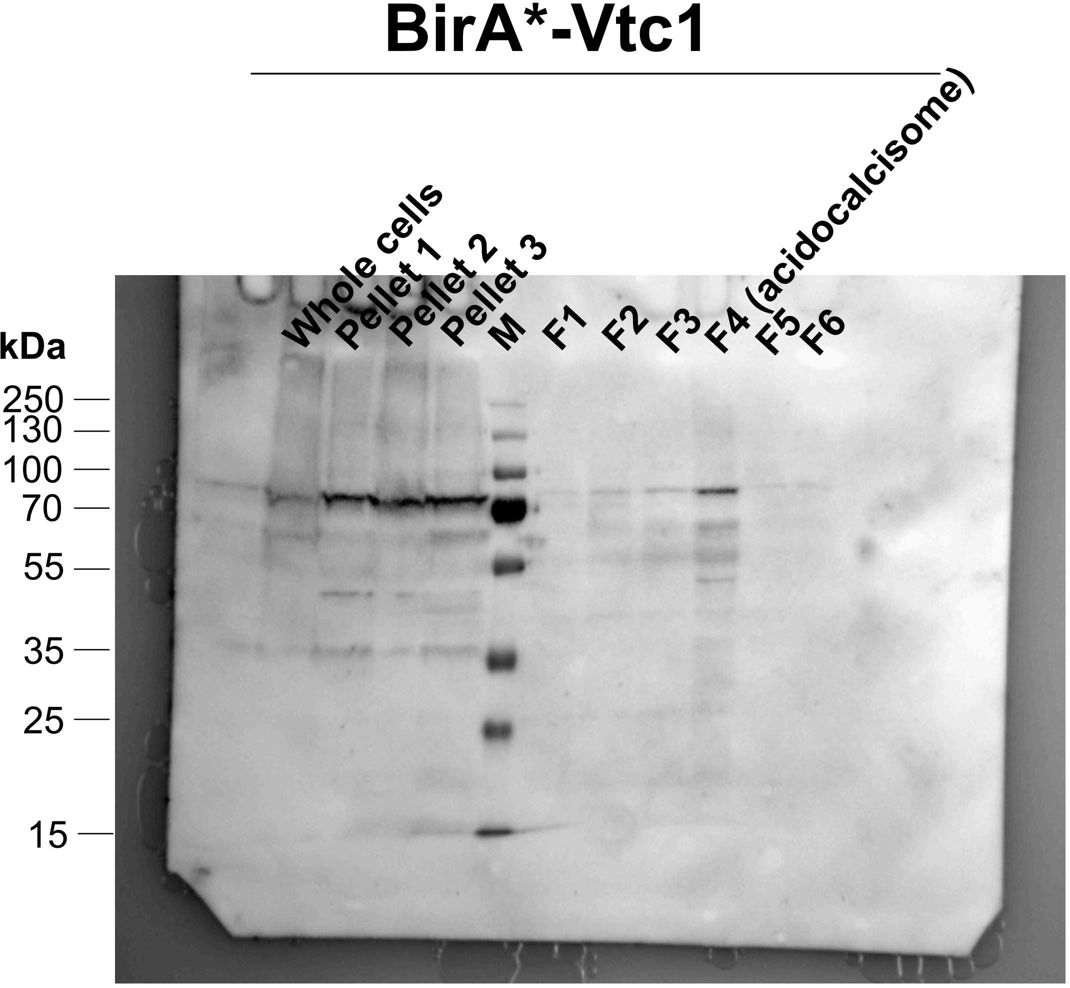

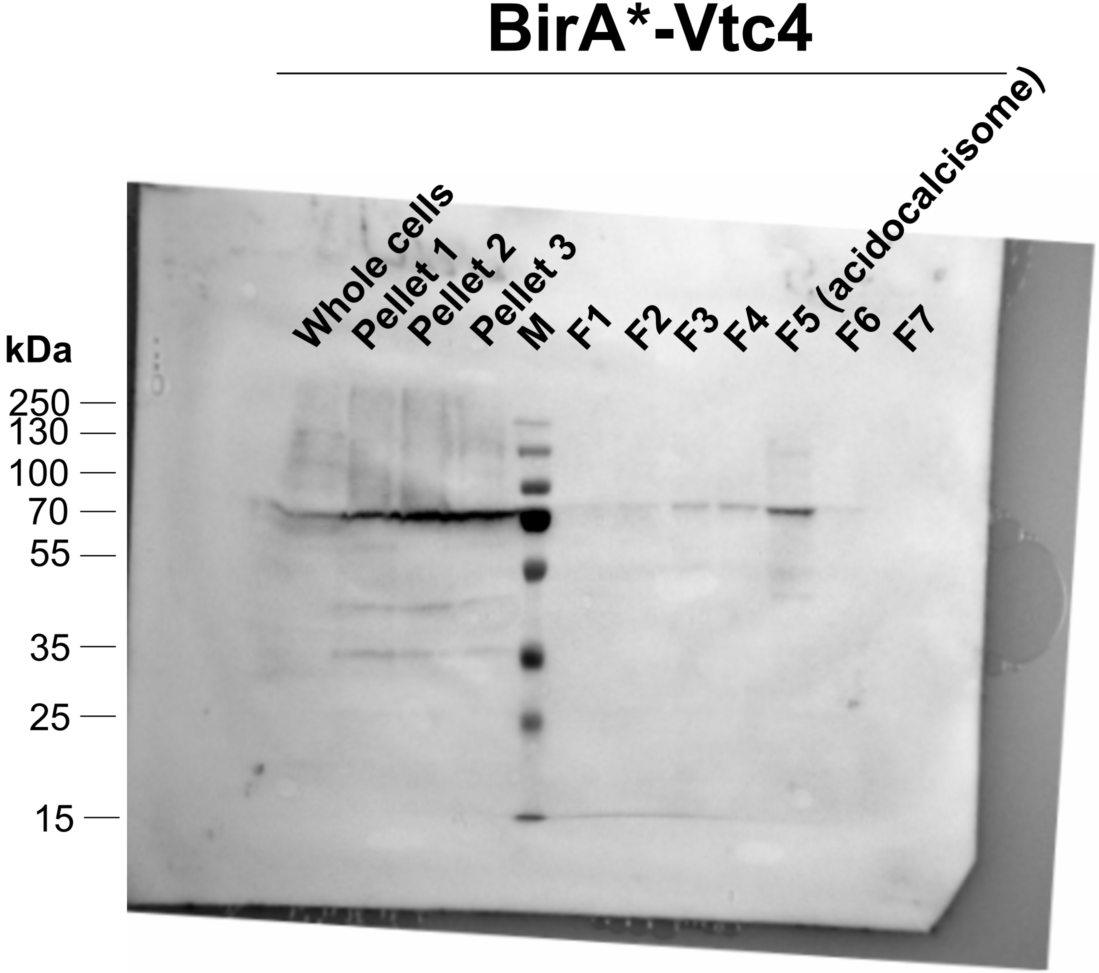

S1 Fig panel H Fluorescence gel

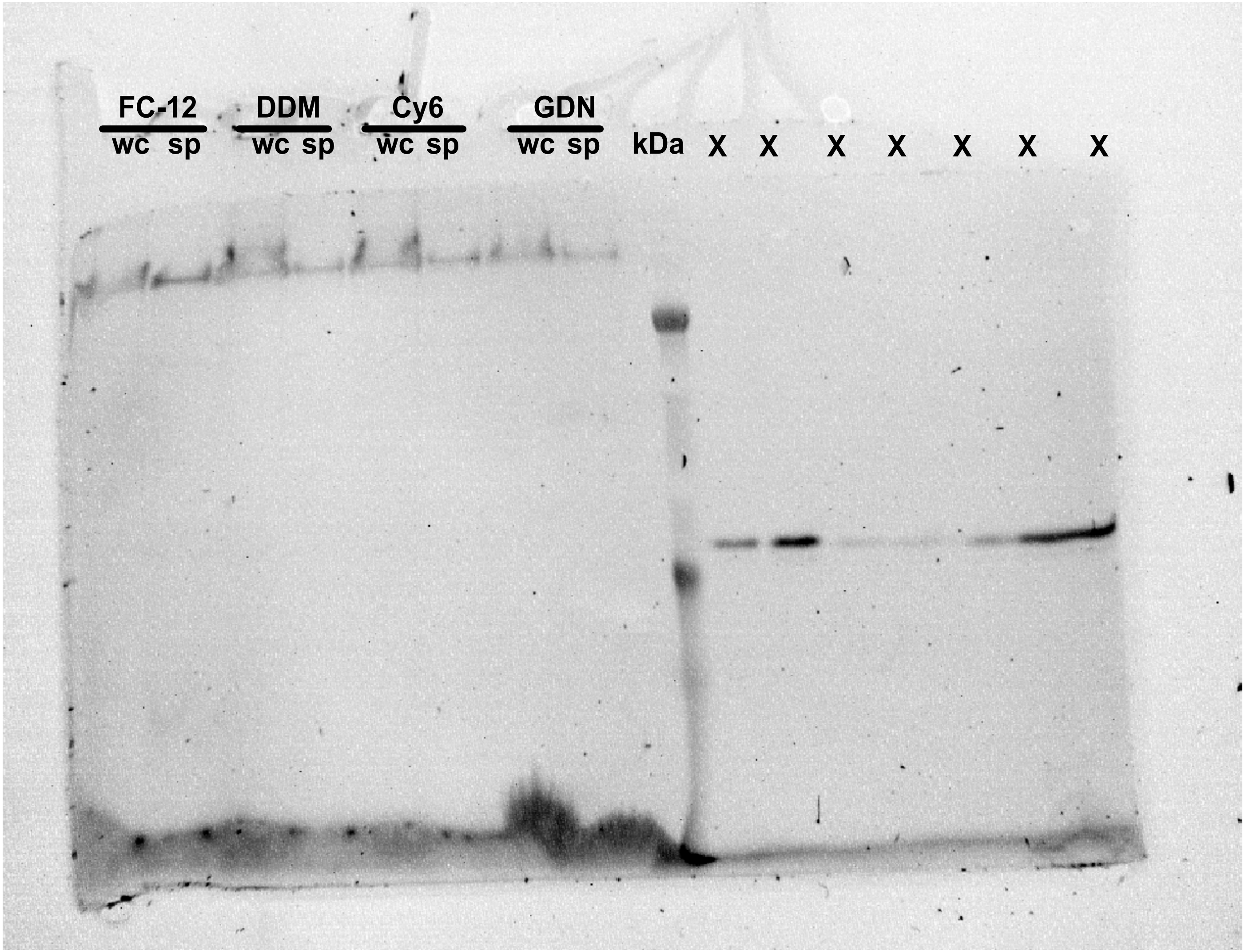

S2 Fig Fluorescence gels

A

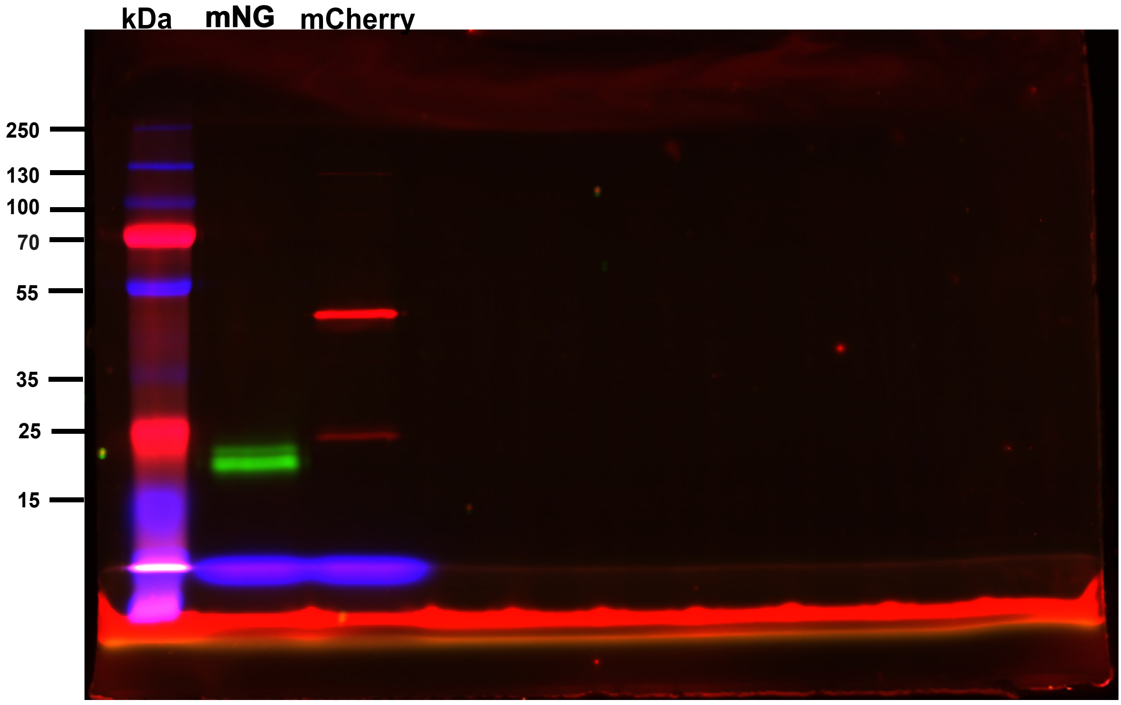

B

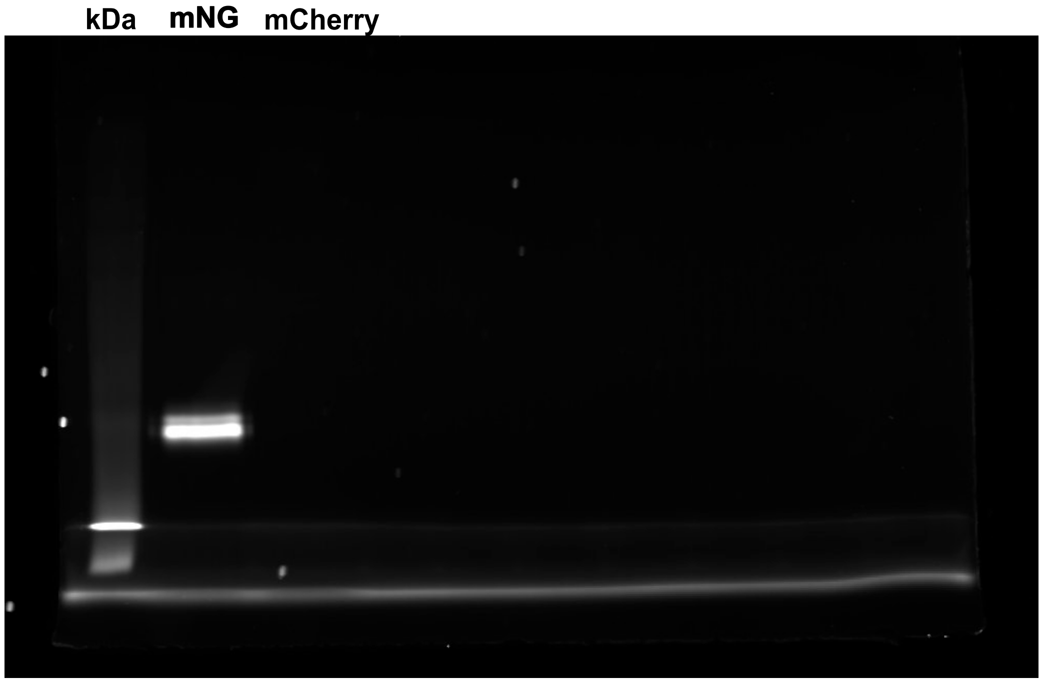

C

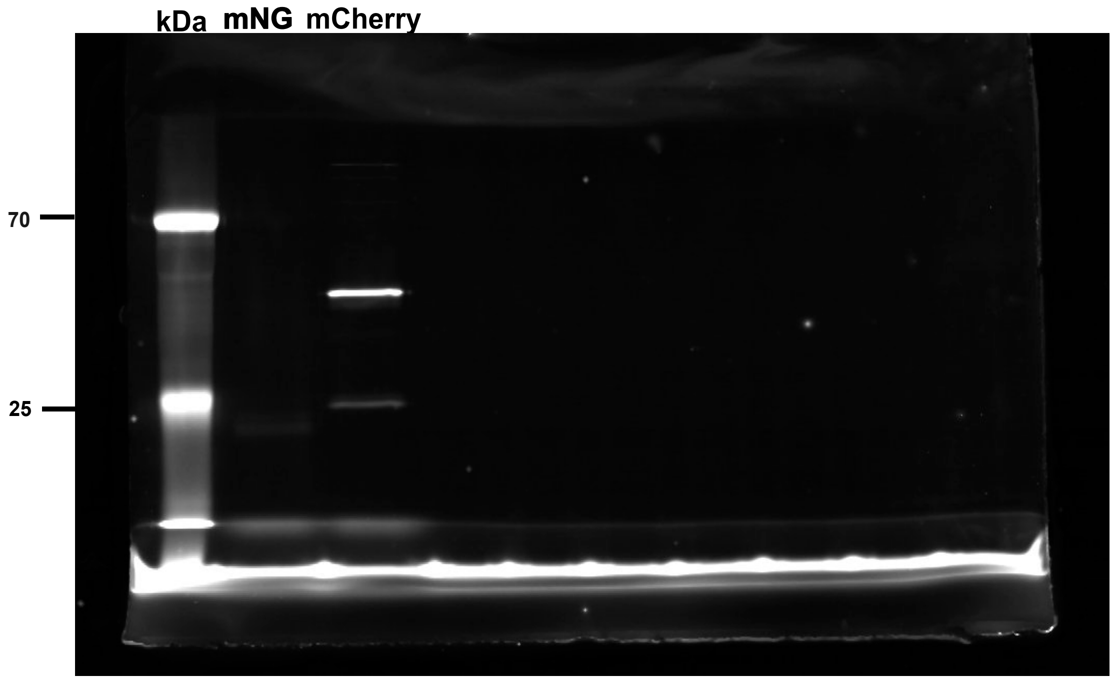

S3 Fig

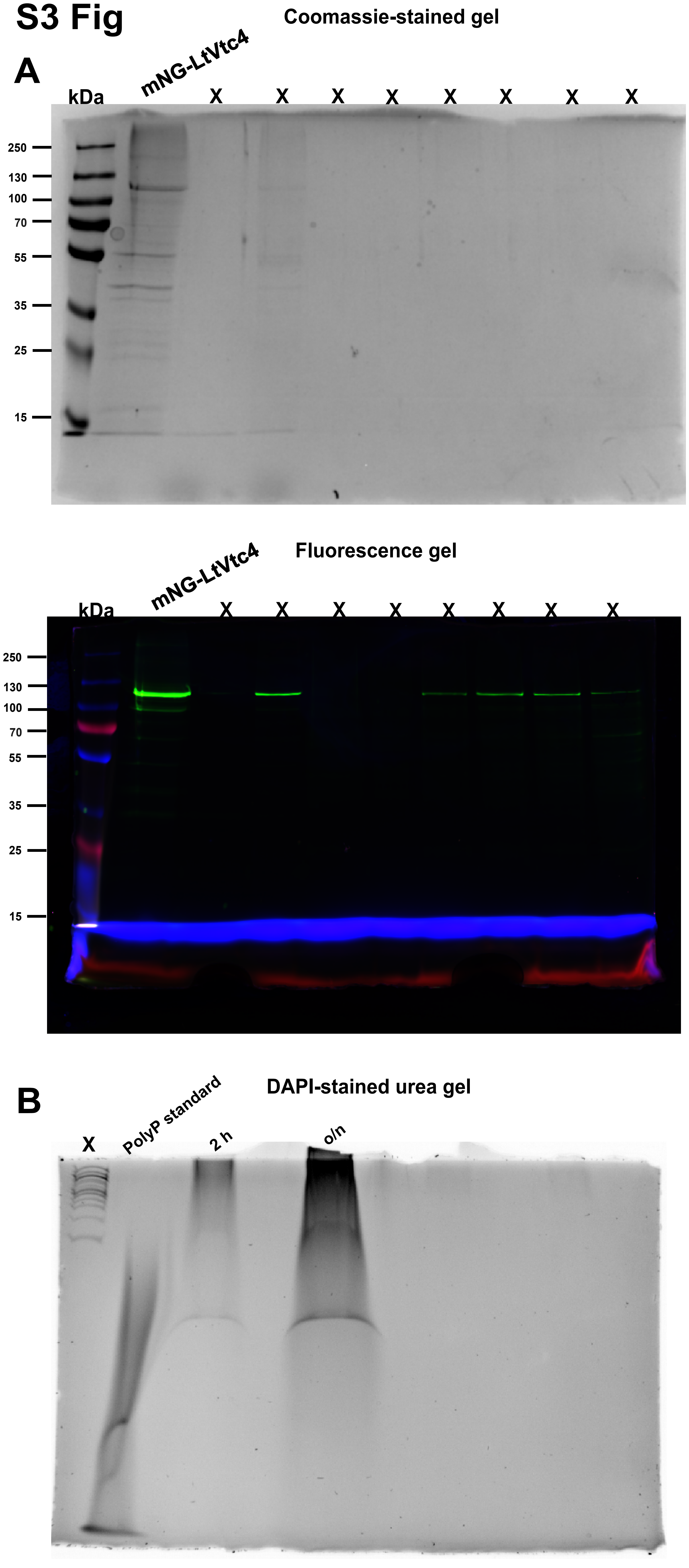
Coomassie-stained gel
A
kDa
mNG-LtVtc4
X
250
130
100
70
55
35
25
15
Fluorescence gel
kDa
mNG-LtVtc4
X
250
130
100
70
55
35
25
15
B
DAPI-stained urea gel
X
PolyP standard
2 h
o/n

S5 Fig Fluorescence gels

A

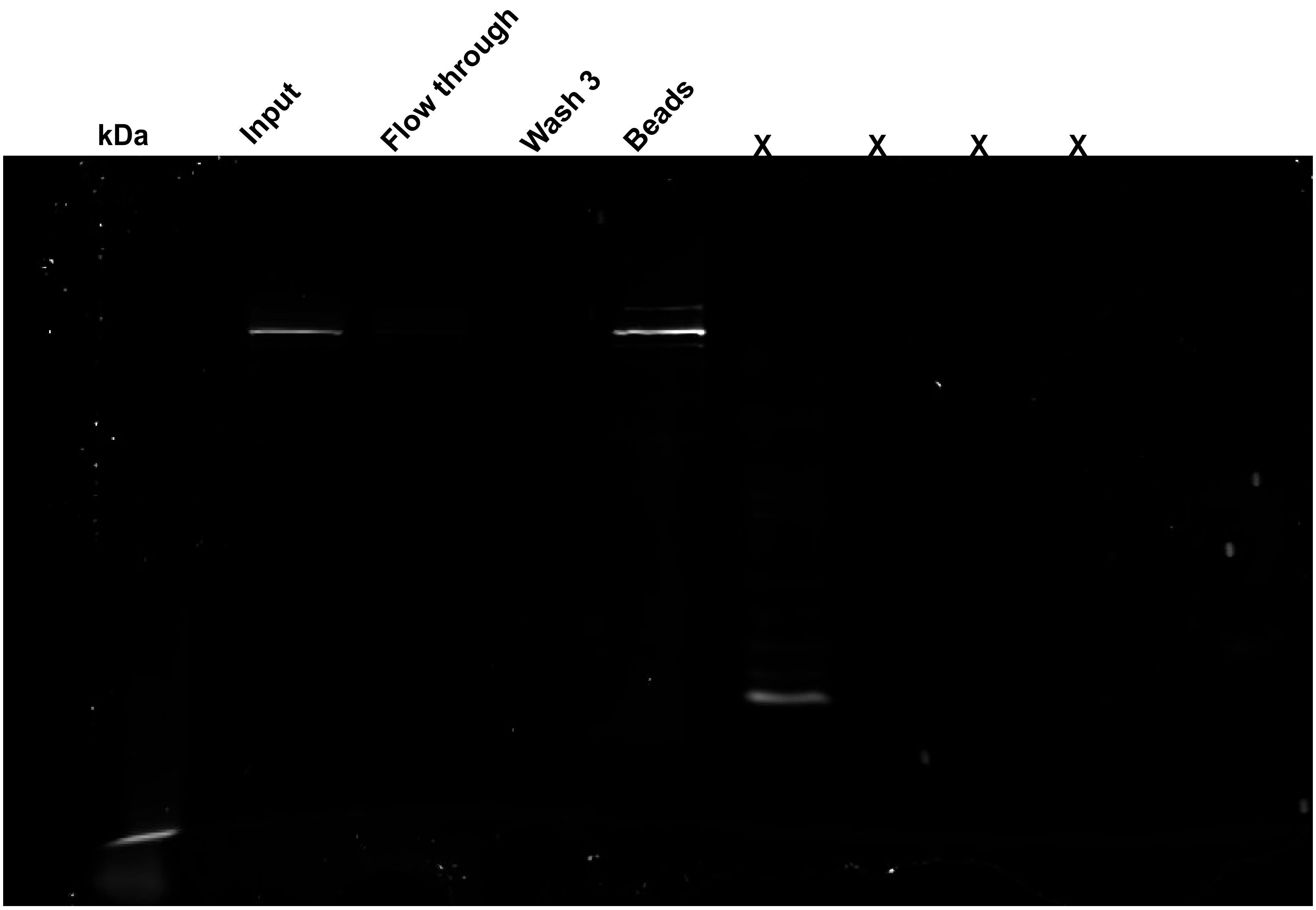

B

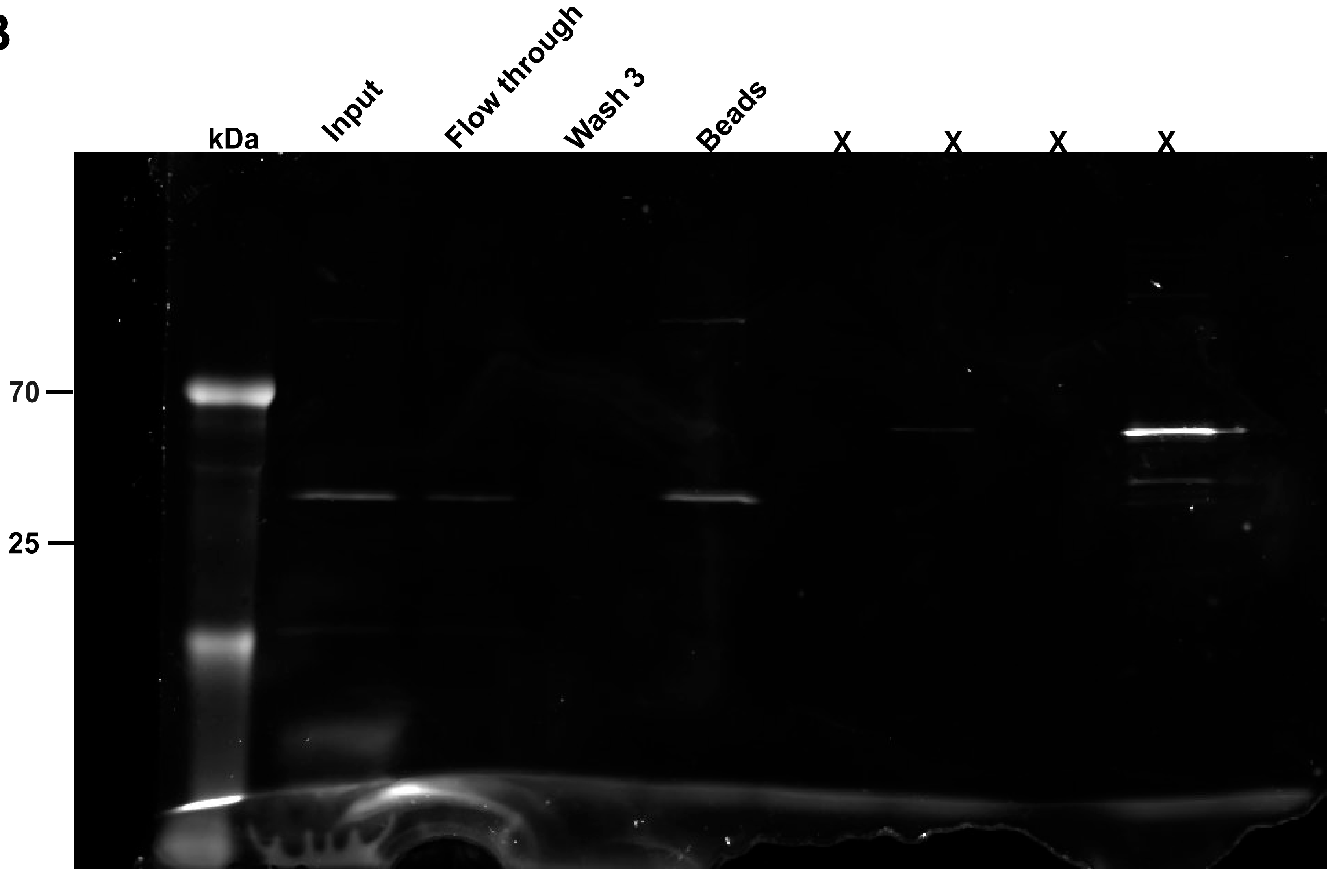

S6 Fig Fluorescence gels

A

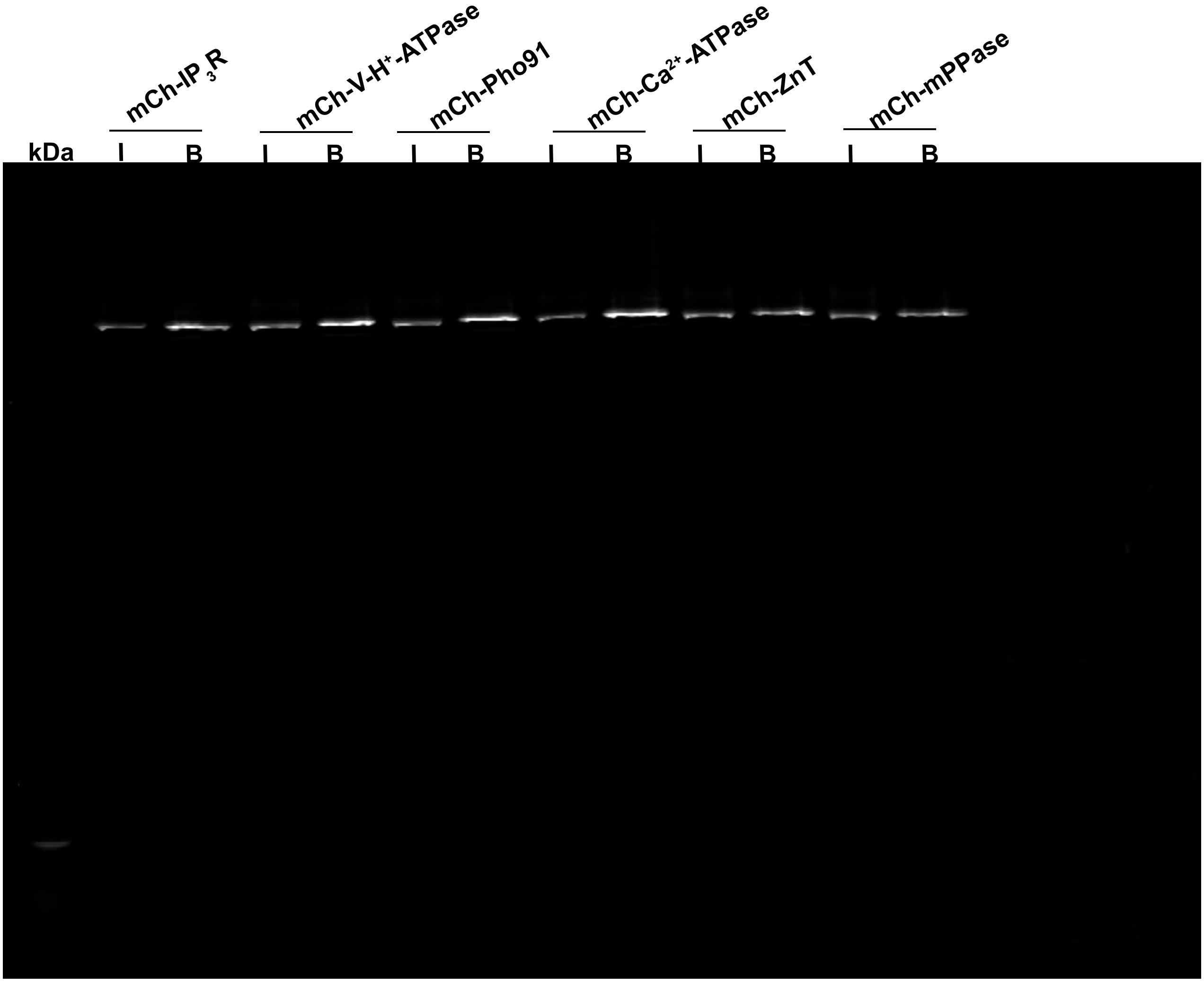

B

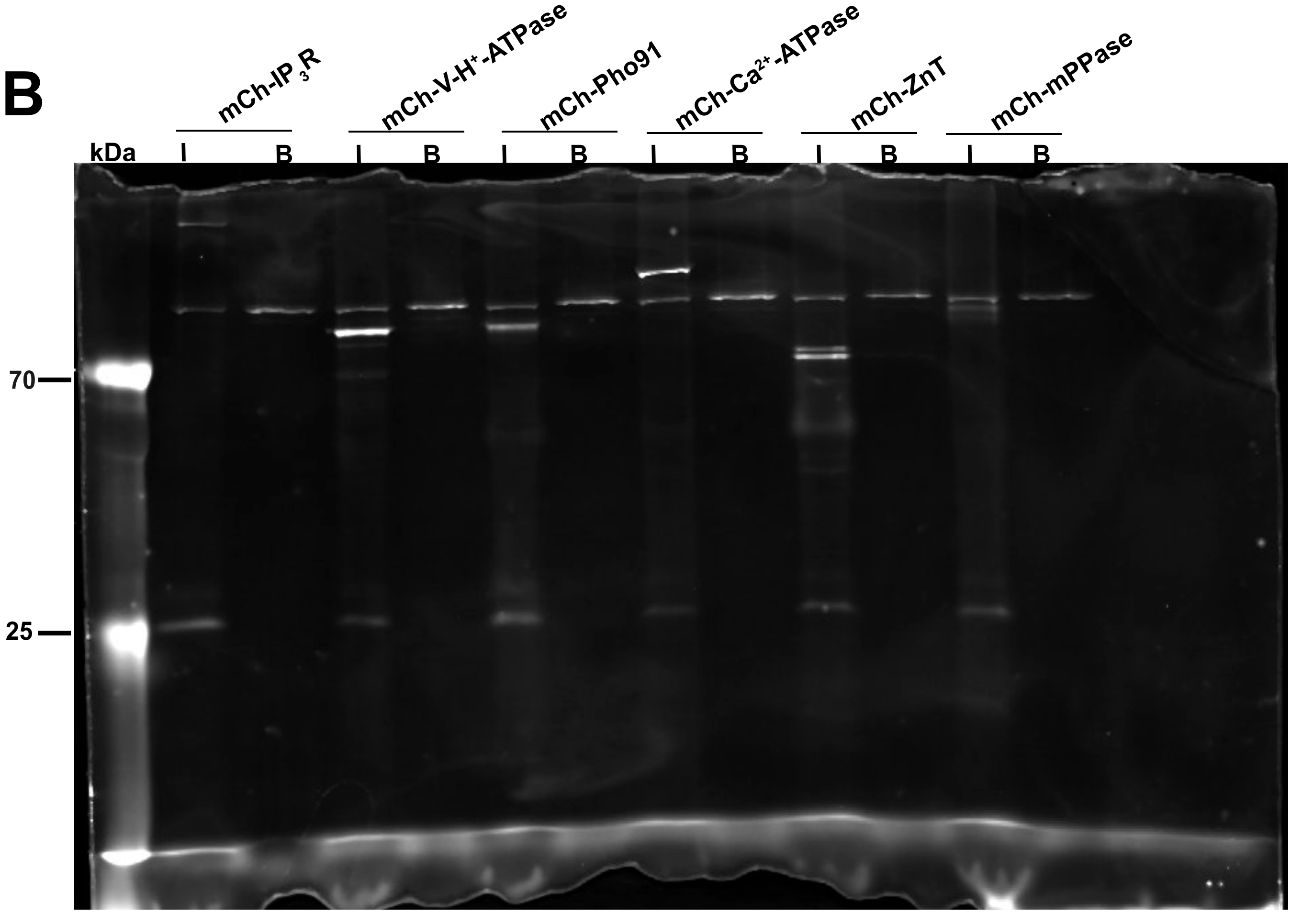

S8 Fig Fluorescence gels

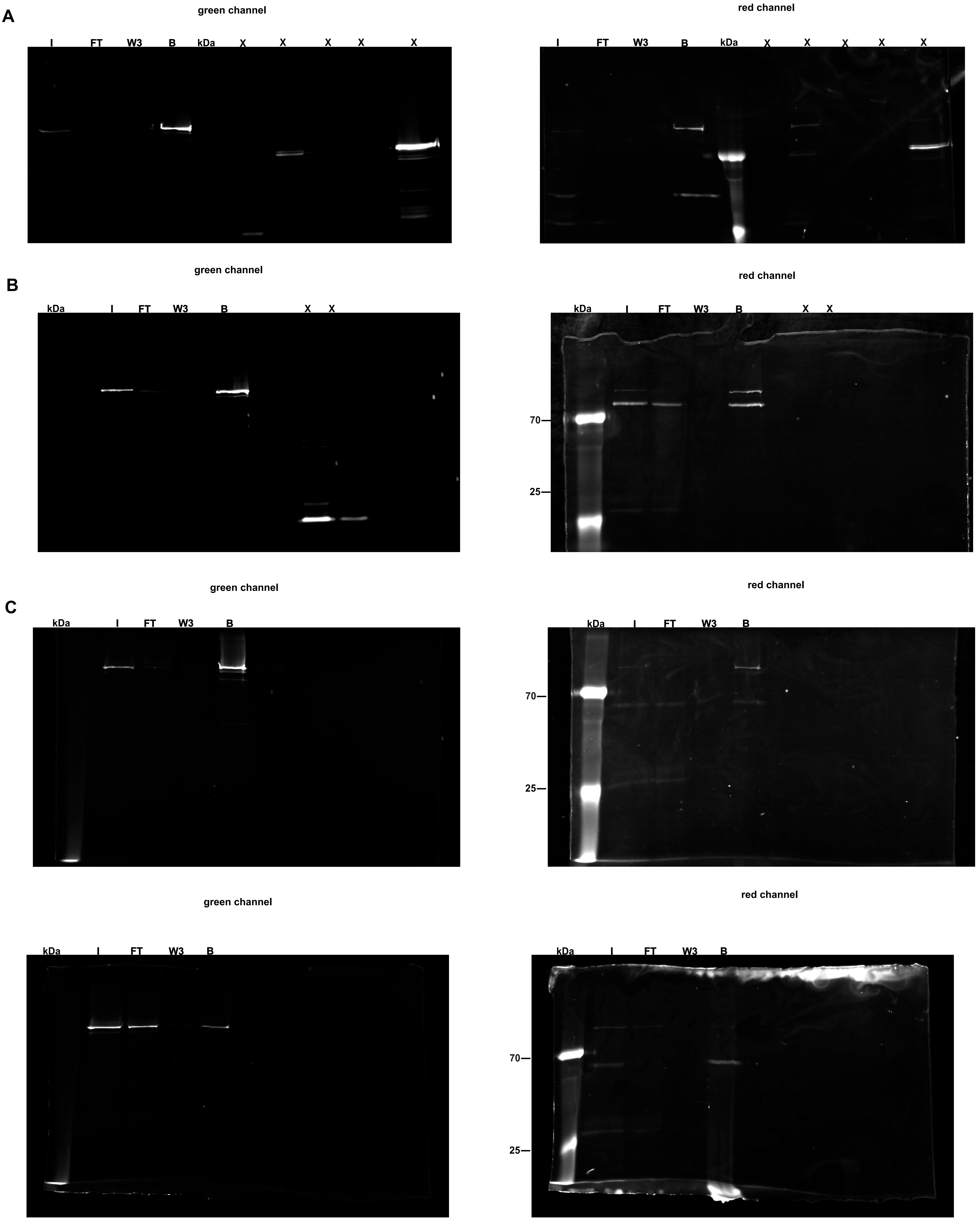

S9 Fig PCR agarose gel

C

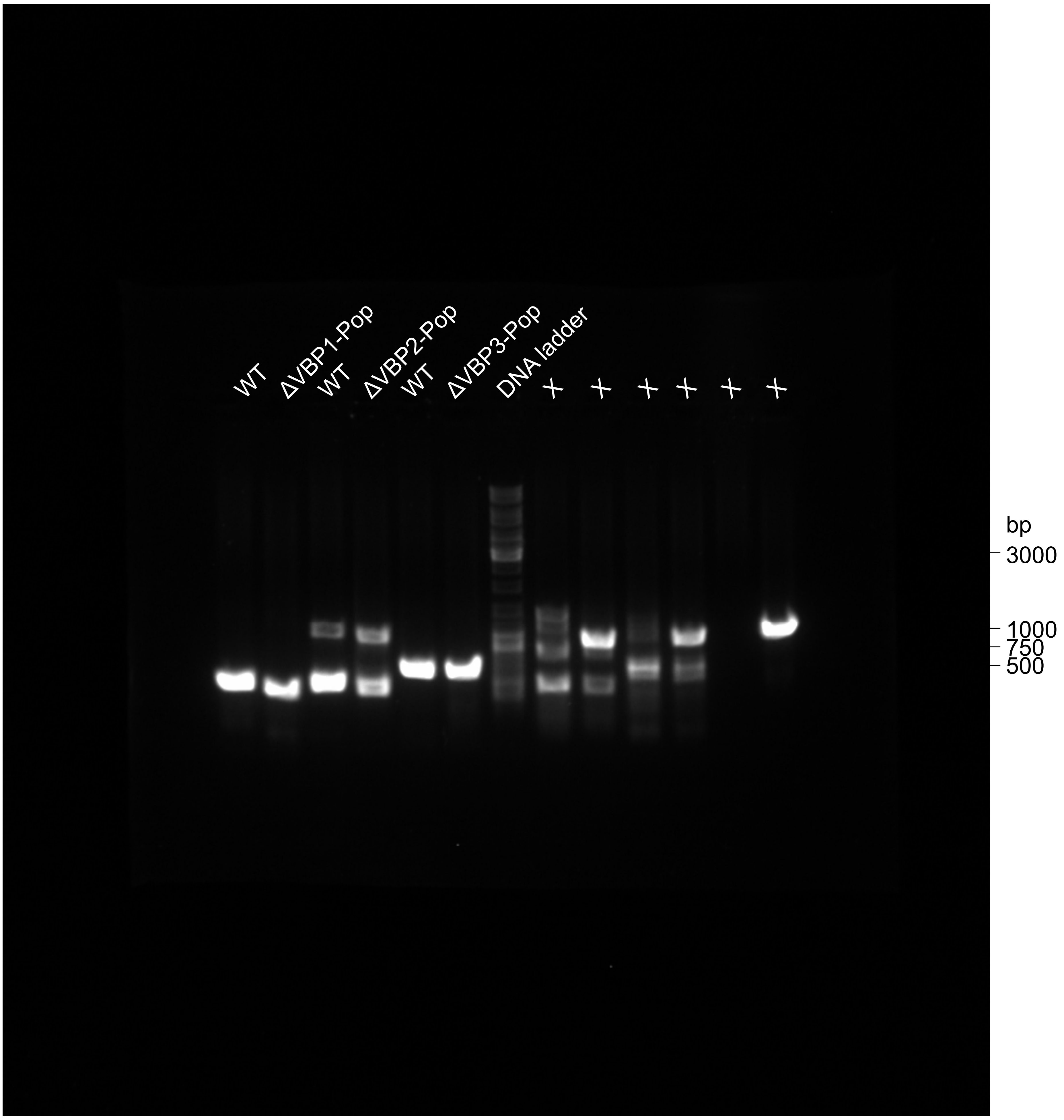

D

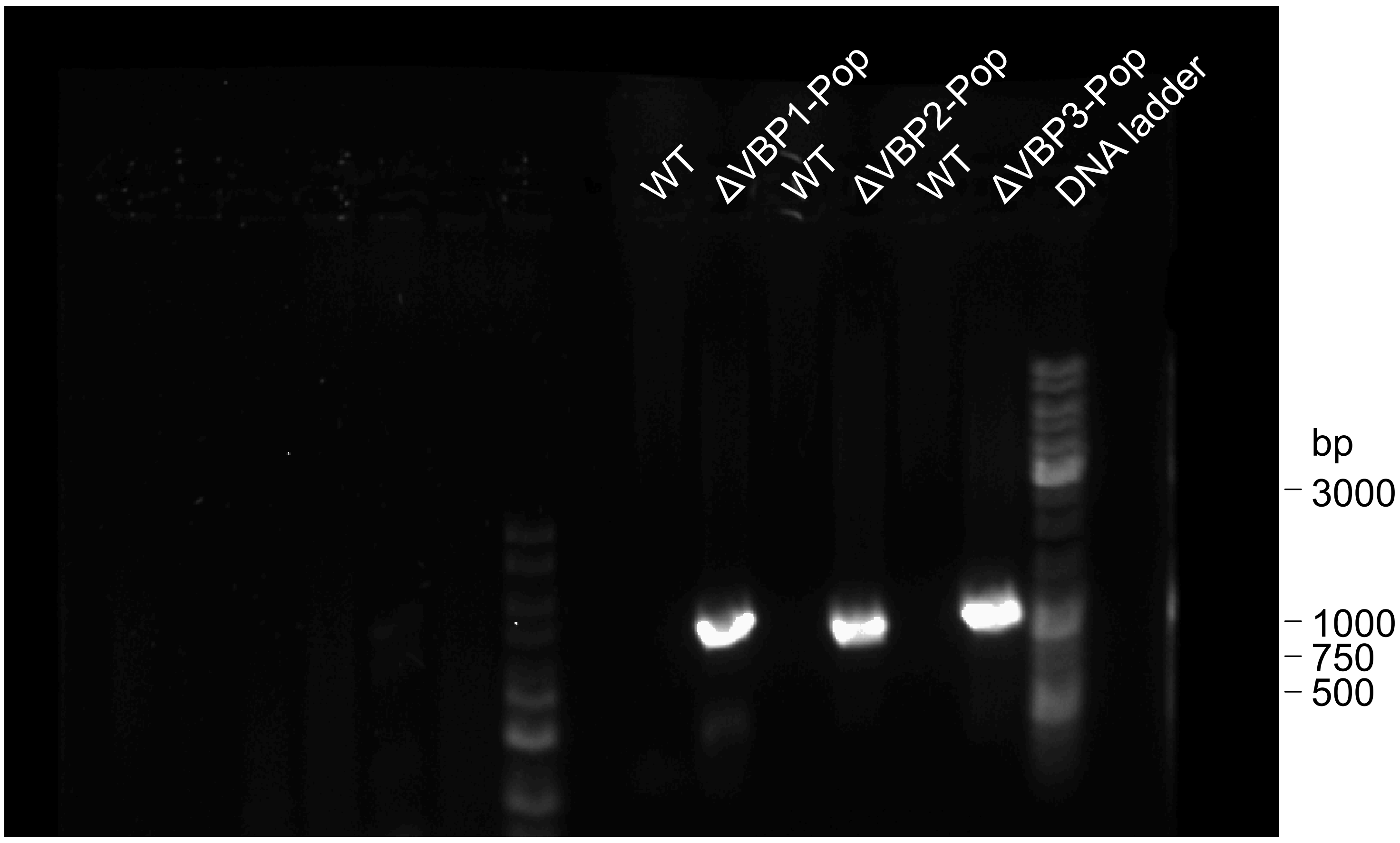

Supplement: S1 Raw Image — (PDF) [file pntd.0014511.s013.pdf]
