# Supplementary material for: Novel binding partners of the Vacuolar Transporter Chaperone (VTC) complex in Acidocalcisomes of Leishmania tarentolae
Source: PLoS Negl Trop Dis. 2026 Jul 13;20(7):e0014511. doi: 10.1371/journal.pntd.0014511 (PMC13375126; doi:10.1371/journal.pntd.0014511)
Supplement: Attachment — Submitted filename: Response to Reviewers_revision2.docx [file pntd.0014511.s024.docx]

Please include the following items when submitting your revised manuscript:

* A letter that responds to each point raised by the editor and reviewer(s). You should upload this letter as a separate file labeled 'Response to Reviewers'. This file does not need to include responses to any formatting updates and technical items listed in the 'Journal Requirements' section below.
* A marked-up copy of your manuscript that highlights changes made to the original version. You should upload this as a separate file labeled 'Revised Manuscript with Track Changes'.
* An unmarked version of your revised paper without tracked changes. You should upload this as a separate file labeled 'Manuscript'.

If you would like to make changes to your financial disclosure, competing interests statement, or data availability statement, please make these updates within the submission form at the time of resubmission. Guidelines for resubmitting your figure files are available below the reviewer comments at the end of this letter.

We look forward to receiving your revised manuscript.

Kind regards,

Rhoel Ramos Dinglasan
Academic Editor
PLOS Neglected Tropical Diseases

Sarman Singh
Section Editor
PLOS Neglected Tropical Diseases

Shaden Kamhawi
co-Editor-in-Chief
PLOS Neglected Tropical Diseases
orcid.org/0000-0003-4304-636XX

Paul Brindley
co-Editor-in-Chief
PLOS Neglected Tropical Diseases
orcid.org/0000-0003-1765-0002

**Additional Editor Comments:**

1- While addressing each reviewer's critique, please pay close attention to a major concern raised by Reviewer 3 with respect to addressing the apparent lack of functional data when revising your manuscript.

**Response:**

We have addressed this concern by adding new functional and localisation data. First, we now show that the purified VTC complex has polyP synthesis activity in vitro, which has been added as Supplementary Figure 3. Second, we generated VBP1, VBP2, and VBP3 knockout population mutants and analysed their effects on LtVtc4 localisation. These new data are presented in Figure 7. Loss of VBP1 reduced LtVtc4 colocalisation with the acidocalcisomal marker mPPase and caused partial redistribution of LtVtc4 towards the kinetoplast region, whereas loss of VBP2 or VBP3 did not show a detectable effect on LtVtc4 localisation under the conditions tested.

2- Please ensure that MIAPE guidelines are followed as there is no data deposition in a recognized international repository. It is important to include gene IDs/Accession numbers for all identified hits in the main text and supplemental data as searchable files.

**Response:**

We have deposited the proteomics raw data in the PRIDE repository under ProteomeXchange accession number PXD075501. We have also added the relevant gene IDs/accession numbers for the identified proteins in the main text and supplementary data. The protein lists from the proteomics datasets are now provided as searchable supplementary tables.

3- Check through the supplemental data to determine relevance, order of discussion, and appropriateness.

**Response:**

We have reviewed the supplemental data for relevance, order, and appropriateness. Supplementary Figure 4 from the original submission has been removed to avoid possible copyright concerns. The remaining supplementary figures and tables have been revised, reordered where necessary, and retained only where they support the results presented in the manuscript.

**Journal Requirements:**

If the reviewer comments include a recommendation to cite specific previously published works, please review and evaluate these publications to determine whether they are relevant and should be cited. There is no requirement to cite these works unless the editor has indicated otherwise.

1) We ask that a manuscript source file is provided at Revision. Please upload your manuscript file as a .doc, .docx, .rtf or .tex. If you are providing a .tex file, please upload it under the item type u2018LaTeX Source Fileu2019 and leave your .pdf version as the item type u2018Manuscriptu2019.

2) We do not publish any copyright or trademark symbols that usually accompany proprietary names, eg ©,  ®, or TM  (e.g. next to drug or reagent names). Therefore, please remove all instances of trademark/copyright symbols throughout the text, including:
- ® on page: 10
- TM on pages: 8, and 10.

3) Please upload all main figures as separate Figure files in .tif or .eps format. For more information about how to convert and format your figure files please see our guidelines: 
<https://journals.plos.org/plosntds/s/figures>

**Reviewers' Comments:**

Reviewer's Responses to Questions

**Key Review Criteria Required for Acceptance?**

As you describe the new analyses required for acceptance, please consider the following:

**Methods**


-Are the objectives of the study clearly articulated with a clear testable hypothesis stated?

-Is the study design appropriate to address the stated objectives?

-Is the population clearly described and appropriate for the hypothesis being tested?

-Is the sample size sufficient to ensure adequate power to address the hypothesis being tested?

-Were correct statistical analysis used to support conclusions?

-Are there concerns about ethical or regulatory requirements being met?

Reviewer #1: The methods are clearly written and to my understanding sufficiently detailed to be reproducible.

**Response:**

We thank the reviewer for this positive assessment.

Reviewer #2: The work described in this manuscript was designed to explore the composition of the VTC complex in the Leishmania tarentolae acidocalcisomes. In pursuing this objective, the authors used several methodologies: mass spectrometry, proximity-dependent biotinylation, immunofluorescence microscopy, endogenous labelling of proteins, and structural predictions. As a result, the authors gathered enough information to provide a plausible composition of the VTC complex consisting of two known subunits (VTC1 and VTC4) and three novel VTC binding partners (VBPs). The experimental results are clear and well-illustrated. The structural analyses and the proposed model for the VTC complex are convincing. In sum, this study is a valuable contribution that may serve as a starting point for future ones.

**Response:**

We thank the reviewer for this positive evaluation of the study and for recognising the value of the combined experimental and structural approaches.

Reviewer #3: The methods used are well described and proper statistical analysis is applied. Information about the source of some reagents (PPase antibodies) is missing.

**Response:**

We thank the reviewer for pointing this out. We have now added the source of the mPPase antibody in the Methods section. The mPPase signal was detected using a rabbit polyclonal anti-TmPPase antibody generated against purified Thermotoga maritima TmPPase, as previously described.

**Results**


-Does the analysis presented match the analysis plan?

-Are the results clearly and completely presented?

-Are the figures (Tables, Images) of sufficient quality for clarity?

Reviewer #1: The experimental results are well presented and the details sufficient to follow the authors' explanations. However, while the experimental model is Leishmania tarentolae, there are frequent references to T. brucei, which was used to query TryTrip database. It is not always easy to follow, which organism is being discussed, as their is a lot of back and forth, sometimes within a paragraph. Thus, it would be helpful to make sure that it is always clear which organism is being presented at any time in the results section.

The forth paragraphs from the introduction (lines 105 to 111) seem from its content something that would be better placed at the beginning of the results section as it already dives into experimental proceedings. While this is ultimately an editorial decision, I would recommend doing so.

Please, note that supplementary figure 3 is currently being discussed before supplementary figure 2. Please, invert the order of these two figures.

**Response:**

We have carefully revised the Results section to make clear when *Leishmania tarentolae* or *Trypanosoma brucei* is being discussed, so that the comparisons between organisms are easier to follow.

The paragraph previously located in the Introduction (lines 105–111) has been moved to the beginning of the Results section and revised to better fit the experimental focus of that section (lines 533–537).

We have checked the order of citation of the supplementary figures and ensured that Supplementary Figure 2 is cited before Supplementary Figure 3 in the revised manuscript.

Reviewer #2: See above

Reviewer #3: The results are well presented, and the figures are of good quality. One supplementary figure (Suppl. Fig. 4) appears as taken from a website. Some supplemental figures are not needed.

**Response:**

We thank the reviewer for this comment. Supplementary Figure 4 has been removed from the revised manuscript. In addition, the remaining supplementary figures have been carefully reviewed and revised to ensure that only those necessary to support the results are included.

**Conclusions**


-Are the conclusions supported by the data presented?

-Are the limitations of analysis clearly described?

-Do the authors discuss how these data can be helpful to advance our understanding of the topic under study?

-Is public health relevance addressed?

Reviewer #1: The results provide sufficient evidence to support the authors' conclusion on how the VTC complex is assembled in kinetoplastids, like L. tarentolae, as summarized in figure 9.

**Response:**

We thank the reviewer for this positive assessment.

Reviewer #2: See above

Reviewer #3: This manuscript reports a study on the components of the vacuolar transporter chaperone (VTC) complex of Leishmania tarentolae. This complex is present in some eukaryotes, has polyphosphate polymerase activity, localizes to vacuoles and acidocalcisomes, and has been well characterized in yeast as possessing 5 subunits (Vtc1-Vtc5). However, only homologs to Vtc1 and the catalytic subunit Vtc4 have been found in trypanosomatids. Using proximity-dependent biotinylation (BioID) the authors found several proteins previously demonstrated to localize to acidocalcisomes in trypanosomatids including 3 putative binding partners, which also localize to acidocalcisomes and interact with LtVtc1 and LtVtc4 in pulldown assays. AlphaFold 3 structural predictions also suggest their interaction with the complex.

**Response:**

We thank the reviewer for this positive assessment.

**Editorial and Data Presentation Modifications**


Use this section for editorial suggestions as well as relatively minor modifications of existing data that would enhance clarity. If the only modifications needed are minor and/or editorial, you may wish to recommend “Minor Revision” or “Accept”.

Reviewer #1: Overall, this is very well structure manuscript. With the exception of the couple of comments in the results section, I have no further comments here.

Reviewer #2: Although the manuscript is well-written, several issues require modification or clarification.
Specific points.

1. Line 142. Pen-Strep likely refers to an antibiotic mixture, but this should be clarified. Also, it should be indicated whether this mixture was used as a common component of the growth media or used specifically at this stage.

**Response:**

The abbreviation Pen-Strep has now been clarified in the Methods section as penicillin–streptomycin (antibiotic mixture), including the concentration used, and it is specified that it is used as a component of the growth medium.

2. Line 198. The generation of the anti-mPPase antibody should be briefly commented on.

**Response:**

The generation of the anti-mPPase antibody is now briefly described in the Methods section under “Acidocalcisome isolation/preparation.”

3. Line 223-224: The sentence "the peptide... recovered" has a cryptic meaning. Please, clarify.

**Response:**

We have clarified this sentence in the revised manuscript. It now reads:

“The peptide digests were centrifuged to pellet the beads, and the supernatant containing the released peptides was collected. Trypsin solution was then added, and the mixture was incubated for 4 h at 37 °C.”

4. Line 245. Please, specify what IP6 is.

**Response:**

IP_6_ is now defined in the revised manuscript at first mention as inositol hexakisphosphate (IP_6_).

5. If DDM is not a standard abbreviation and may have different meanings, please indicate (first time it is mentioned) that it corresponds to dodecyl-beta-maltoside (as it is indicated in the supplementary fig 1 legend). This compound is extensively used in this work and merits avoiding any confusion about its nature. The same apply for FC-12 (Fos-choline-12).

**Response:**

DDM and FC-12 are now defined at first mention in the revised manuscript. DDM is specified as *n*-dodecyl-β-D-maltoside, and FC-12 as Fos-choline-12.

6. Line 261. Please, specify what "fluorescent protein marker" was used.

**Response:**

We have revised the text to specify the protein marker used. The revised manuscript now identifies it as PageRuler Plus Prestained Protein Ladder (26619, Thermo Fisher Scientific), which was detected in the 638 nm channel.

7. Line 310. To facilitate the identification of orthologs for LtVtc1 and LtVtc4 in other kinetoplastids, the inclusion (here or in a previous section) of the TriTryDB gene IDs for these Lt genes would be valuable.

**Response:**

The TriTryDB gene IDs for LtVtc1 and LtVtc4 have now been added to the manuscript to facilitate the identification of orthologs in other kinetoplastids.

8. Lines 343-344. It is unclear what “1240 out of the 3160 identified proteins are unique hits” means. Could you rephrase the sentence for clarity?
Moreover, an Excel file with the proteins identified in each one of the four datasets would be a valuable resource. Therefore, this reviewer encourages authors to provide this information within a supplementary file.

**Response:**

The sentence has been rephrased for clarity. It now reads:

“Affinity purification of the biotinylated proteins followed by liquid chromatography–mass spectrometry (LC–MS) resulted in a total of 3,160 protein identifications across four datasets, corresponding to 1,240 non-redundant (unique) protein hits (Fig 2A).”

The lists of proteins identified in the four datasets have now been included as searchable supplementary Excel tables and are provided as Supplementary Tables S2–S5.

9. Line 365. How are poly-P binding proteins identified? Is there any structural feature that was used to make the selection? Please, clarify this.

**Response:**

We have clarified that polyP-binding proteins were defined based on previously reported polyP–protein interactions in trypanosomatids (Negreiros et al., 2018), rather than structural motif prediction.

10. Lines 609-610. The article quoted as reference 60 deals with a T. cruzi WD-repeat containing protein. Please, check if referring to L. major in this sentence is appropriate.

**Response:**

The sentence has been revised to avoid species-specific reference. It now reads:

“In trypanosomatids, WD-repeat β-propeller proteins contribute to cell cycle control and signaling.”

11. The authors should consider depositing the proteomics raw data in a repository like PRIDE or similar.

**Response:**

The proteomics raw data have been deposited in the PRIDE repository (ProteomeXchange Consortium) under accession number PXD075501.

Reviewer #3: Major points: The work provides confirmation for the acidocalcisome localization of several proteins and describes new putative components of the VTC complex in Leishmania. However, there is no evidence presented on the enzymatic activity of the complex, on how this is affected by the three putative binding partners, or on the essentiality of the new components for the life cycle of L. tarentolae. Experimental evidence on these aspects, as well as some corrections, needs to be provided in order to substantiate the conclusions and improve the quality of this work.

**Response:**

We have now added the polyP synthesis activity of the purified VTC complex in Supplementary Figure 3. To address the roles of the three VBPs, we performed knockout experiments and analysed their effects on Vtc4 localisation (Figure 7). Knockout of VBP2 or VBP3 did not affect Vtc4 localisation to acidocalcisomes, whereas VBP1 knockout reduced Vtc4 colocalisation with acidocalcisomes. Interestingly, VBP1 knockout also led to partial redistribution of LtVtc4 towards the kinetoplast region.

Minor points:

1. Fig. 1B shows bands of the same size for mNG-VTC1 and mNG-VTC4. Is this right? Fig. 1D shows co-localization with mPPase antibodies. Which is the source of these antibodies? Is this tagged PPase?

**Response:**

Fig. 1B: Yes, this is expected. In Fig. 1B, PCR was used to verify the correct integration of the mNG tagging cassette. The primers flank the entire cassette, which is identical in size for both VTC1 and VTC4, resulting in PCR products of the same length.

Fig. 1D: The mPPase signal was detected using a rabbit polyclonal anti-TmPPase antibody generated against purified *Thermotoga maritima* TmPPase and has been described previously (Liu et al., 2025, PLoS ONE 20(5): e0322756). The generation of the anti-mPPase antibody is now briefly described in the Methods section under “Acidocalcisome isolation/preparation.”

2. Fig. 2. An Excel file with the list of proteins identified in different experiments will be appropriate as supplemental information.

**Response:**

The lists of proteins identified in the four datasets have now been included as supplementary material and are provided as Supplementary Tables S2–S5.

3. Table 1. There are no Vacuolar-P-type ATPases. The name vacuolar should be used only for the V-ATPases. V-ATPases are different from P-type ATPases.

**Response:**

The terminology has been corrected. “Vacuolar-P-type ATPases” has been changed to “P-type Ca²⁺-ATPase” in the revised Table 1.

4. Suppl. Fig. 1A shows bands of the same size for BirA+-VTC1 and BirA+-VTC4. Is this right? Fig. 1H is not explained. Loading controls are missing in the western blots and should be provided. Which is the source of TmPPase antibody?

**Response:**

Suppl. Fig. 1A: Yes, this is expected. In Suppl. Fig. 1A, PCR was used to confirm correct genomic integration of the BirA* tagging cassette. The same primer pair flanking the cassette was used for both cell lines. Since the BirA* cassette is identical in size for BirA*-VTC1 and BirA*-VTC4, the resulting PCR products are of the same length. Importantly, this PCR product size reflects the length of the integrated cassette and does not correspond to the size of the encoded fusion proteins. In contrast, Suppl. Fig. 1B shows Western blot analysis of the expressed fusion proteins. Here, BirA*-VTC1 and BirA*-VTC4 migrate at different apparent molecular weights, consistent with the different sizes of the VTC1 and VTC4 proteins.

Suppl. Fig. 1H: This figure is described in the corresponding legend and is also referenced and explained in the main text under “Identification of proteins in close proximity to VTC.”

Suppl. Fig. panel B and C: Ponceau S staining of the membrane has now been included below the western blots as a loading control to confirm comparable protein loading.

Suppl. Fig. 1F: The rabbit polyclonal anti-TmPPase antibody was generated against purified *Thermotoga maritima* TmPPase, as previously described (Liu et al., 2025, *PLoS ONE* 20(5): e0322756). The generation of the anti-mPPase antibody is now briefly described in the Methods section under “Acidocalcisome isolation/preparation.”

5. Suppl. Fig. 2 is not mentioned in the text.

**Response:**

## Suppl. Fig. 2: This figure is referenced in both the Methods section (Pulldown assays) and in the Results section (Proximal acidocalcisome proteins and novel interactors of the VTC Complex in *Leishmania tarentolae).*

6. Suppl. Fig. 4. Is this taken from a website? Do you have permission to reproduce it?

**Response:**

To avoid any potential copyright concerns, Supplementary Figure 4 has been removed from the revised manuscript.

7. Suppl. Figs. 7 and 10. These figures are not necessary.

**Response:**

Supplementary Figures 7 and 11 (Figure 10 in the original submission) have been revised and retained because they provide supporting information relevant to the results presented.

8. Suppl. Tables 2-5 can be replaced with an Excel file listing all the proteins identified without attributing localization, or polyphosphorylation, as the attribution indicates only a possibility that for the most part has not been validated.

**Response:**

The annotations referring to localisation and polyphosphorylation have been removed from Supplementary Tables 2–5 (now Supplementary tables 6-9) to avoid attributing functions or localisations that have not been experimentally validated.

**Summary and General Comments**


Use this section to provide overall comments, discuss strengths/weaknesses of the study, novelty, significance, general execution and scholarship. You may also include additional comments for the author, including concerns about dual publication, research ethics, or publication ethics. If requesting major revision, please articulate the new experiments that are needed.

Reviewer #1: The manuscript titled "Novel Binding Partners of the Vacuolar Transporter Chaperone (VTC) complex in Acidocalcisomes of Leishmania tarentolae" is technically very dense work. It provides sufficient details to insure that experimental execution was successful and that presented results hold up to interpretation. The only weakness I am currently seeing is that the manuscript it not always easy to follow and may be a challenge for an interested reader from the Leishmania field, who is not that deep into proteomics. While I understand that it is challenging in a technically dense study to be generally comprehensible, I believe it would benefit the study to be clearer on when the authors refer to L. tarentolae and when to T. brucei. Make sure that it is clear, when you target the whole acidocalcisome and when only the protein(s) in an assay. Make it clearer to the reader what the GO term analysis is telling us. It may also be worth to include in the discussion what the authors' findings mean to the field of kinetoplastid research; why does it help the field to know the components of the VTC complex?

Beyond a bit more clarity, I do not see any needs for more experiments.

**Response:**

We thank the reviewer for the positive assessment of our work and for the constructive suggestions aimed at improving clarity and accessibility.

To address these points:

- We have carefully revised the manuscript to clearly distinguish when referring to *Leishmania tarentolae* and when referring to *Trypanosoma brucei*, in order to avoid confusion.
- We have clarified throughout the text when experiments target the entire acidocalcisome versus specific proteins within an assay.
- The description of the Gene Ontology (GO) term analysis has been expanded in the section “Identification of proteins in close proximity to the VTC complex” to better explain what the enrichment analysis reveals and how it supports the biological interpretation of the BioID data.
- The Discussion has been strengthened to better contextualise our findings within the field of kinetoplastid research. In particular, we now emphasise that defining the interaction partners of the VTC complex provides a reference framework for understanding its organisation in kinetoplastids, highlights potential species-specific adaptations distinct from the yeast architecture, and expands the molecular context in which the VTC complex operates within acidocalcisomes.

Reviewer #2: See above

Reviewer #3: The manuscript presents results showing proximity-dependent biotinylation (BioID) assays of VTC subunits that identify 3 putative binding partners, which localize to acidocalcisomes and interact with LtVtc1 and LtVtc4 in pulldown assays. AlphaFold 3 structural predictions also suggest their interaction with the complex. However, there is no evidence of the polyphosphate polymerase activity of the complex or that there is a complex and of the role of the binding partners in the activity of the complex.
The results are interesting. However, evidence on the enzymatic activity of the complex, on the role of putative binding partners in the formation of a complex, and on their effect on its activity will greatly enhance the value of the paper.

**Response:**

We thank the reviewer for these valuable comments.

To further investigate the role of the identified VTC binding partners, we performed additional experiments analysing the localisation of LtVtc4 in knockout population mutants lacking VBP1, VBP2, or VBP3. These new data have been included in the revised manuscript (Fig. 7)

Using confocal microscopy and colocalisation analysis with the acidocalcisomal marker mPPase, we observed that deletion of VBP1 resulted in reduced colocalisation of LtVtc4 with acidocalcisomes, together with partial redistribution towards the kinetoplast region. In contrast, deletion of VBP2 or VBP3 did not produce a detectable effect on LtVtc4 localisation under the conditions tested. Statistical analysis of PCC values confirmed a significant effect, specifically in ∆VBP1-Pop cells.

These findings provide evidence that VBP1 contributes to the spatial organisation of LtVtc4 and support a role for VTC-associated proteins in the organisation of the acidocalcisomal VTC environment. In contrast, VBP2 and VBP3 did not show a detectable effect on LtVtc4 localisation under the conditions tested. They may contribute through structural or regulatory interactions within the complex, and their precise roles remain to be determined. Together with the BioID datasets, pulldown assays, and AlphaFold3 structural predictions, these results strengthen the proposed interaction framework surrounding the kinetoplastid VTC complex and establish a basis for future structural studies.

PLOS authors have the option to publish the peer review history of their article ([what does this mean?](https://track.editorialmanager.com/CL0/https:%2F%2Fjournals.plos.org%2Fplosntds%2Fs%2Feditorial-and-peer-review-process%23loc-peer-review-history/1/010f019bd1fe7716-ba0a8d3c-2ac2-4e9c-8dad-d56a5ef104a3-000000/UCPMUPfsVIVhkIhT6gvdYId5In0OaMlksW3-i01vw_8=246)). If published, this will include your full peer review and any attached files.


If you choose “no”, your identity will remain anonymous but your review may still be made public.


**Do you want your identity to be public for this peer review?** For information about this choice, including consent withdrawal, please see our [Privacy Policy](https://track.editorialmanager.com/CL0/https:%2F%2Fwww.plos.org%2Fprivacy-policy/1/010f019bd1fe7716-ba0a8d3c-2ac2-4e9c-8dad-d56a5ef104a3-000000/qU0sTa2MDDFiv9Bpg0oBptqUOkqiU1QdTeD90ObjDaw=246).

Reviewer #1: No

Reviewer #2: No

Reviewer #3: No
