# Supplementary material for: Novel binding partners of the Vacuolar Transporter Chaperone (VTC) complex in Acidocalcisomes of Leishmania tarentolae
Source: PLoS Negl Trop Dis. 2026 Jul 13;20(7):e0014511. doi: 10.1371/journal.pntd.0014511 (PMC13375126; doi:10.1371/journal.pntd.0014511)
Supplement: Attachment — Submitted filename: Response_to_Reviewers_Revision2_auresp_2.docx [file pntd.0014511.s025.docx]

Please include the following items when submitting your revised manuscript:

* A letter that responds to each point raised by the editor and reviewer(s). You should upload this letter as a separate file labeled 'Response to Reviewers'. This file does not need to include responses to any formatting updates and technical items listed in the 'Journal Requirements' section below.

* A marked-up copy of your manuscript that highlights changes made to the original version. You should upload this as a separate file labeled 'Revised Manuscript with Track Changes'.

* An unmarked version of your revised paper without tracked changes. You should upload this as a separate file labeled 'Manuscript'.

If you would like to make changes to your financial disclosure, competing interests statement, or data availability statement, please make these updates within the submission form at the time of resubmission. Guidelines for resubmitting your figure files are available below the reviewer comments at the end of this letter.

As the corresponding author, your ORCID iD is verified in the submission system and will appear in the published article. PLOS supports the use of ORCID, and we encourage all coauthors to register for an ORCID iD and use it as well. Please encourage your coauthors to verify their ORCID iD within the submission system before final acceptance, as unverified ORCID iDs will not appear in the published article. Only the individual author can complete the verification step; PLOS staff cannot verify ORCID iDs on behalf of authors.

We look forward to receiving your revised manuscript.

Kind regards,

Rhoel Ramos Dinglasan

Academic Editor

PLOS Neglected Tropical Diseases

Sarman Singh

Section Editor

PLOS Neglected Tropical Disease

Shaden Kamhawi
co-Editor-in-Chief

PLOS Neglected Tropical Diseases
orcid.org/0000-0003-4304-636XX

Paul Brindley
co-Editor-in-Chief
PLOS Neglected Tropical Diseases
orcid.org/0000-0003-1765-0002

**Reviewers' comments:**

Reviewer's Responses to Questions

**Key Review Criteria Required for Acceptance?**

As you describe the new analyses required for acceptance, please consider the following:

**Methods**

-Are the objectives of the study clearly articulated with a clear testable hypothesis stated?

-Is the study design appropriate to address the stated objectives?

-Is the population clearly described and appropriate for the hypothesis being tested?

-Is the sample size sufficient to ensure adequate power to address the hypothesis being tested?

-Were correct statistical analysis used to support conclusions?

-Are there concerns about ethical or regulatory requirements being met?

Reviewer #1: No further comments

Reviewer #2: As stated in my previous report, this study is well-designed and well-executed. The objectives were clearly articulated. Correct statistical analyses were used.

Reviewer #3: Methods are acceptable

**Results**

-Does the analysis presented match the analysis plan?

-Are the results clearly and completely presented?

-Are the figures (Tables, Images) of sufficient quality for clarity?

Reviewer #1: No further comments

Reviewer #2: The results are clear and exhaustive. The figures, tables, and supplementary materials are of sufficient quality.

Reviewer #3: Authors addressed most of the concerns

**Conclusions**

-Are the conclusions supported by the data presented?

-Are the limitations of analysis clearly described?

-Do the authors discuss how these data can be helpful to advance our understanding of the topic under study?

-Is public health relevance addressed?

Reviewer #1: No further comments

Reviewer #2: The conclusions are based on solid experimental data, and limitations are clearly described. Also, the authors comment on the usefulness of this study for future work.

Reviewer #3: Yes, conclusions support the data

**Editorial and Data Presentation Modifications?**

Use this section for editorial suggestions as well as relatively minor modifications of existing data that would enhance clarity. If the only modifications needed are minor and/or editorial, you may wish to recommend “Minor Revision” or “Accept”.

Reviewer #1: No further comments

Reviewer #2: I agree with the way the authors have addressed my previous suggestions. Moreover, new experiments were added that increase the relevance of this work. No modifications are needed.

Reviewer #3: See comments under the Summary section

**Summary and General Comments**

Use this section to provide overall comments, discuss strengths/weaknesses of the study, novelty, significance, general execution and scholarship. You may also include additional comments for the author, including concerns about dual publication, research ethics, or publication ethics. If requesting major revision, please articulate the new experiments that are needed.

Reviewer #1: Accept

**Response:**

We thank the reviewer for the careful evaluation of our manuscript and for the positive recommendation for acceptance.

Reviewer #2: This study is a valuable contribution to our understanding of the composition of the VTC complex in the Leishmania tarentolae acidocalcisomes. Moreover, it contains structural predictions that may guide future studies. The methodologies described may be of value for other researchers.

**Response:**

We thank the reviewer for the positive assessment of our work and for the constructive comments provided during the review process.

Reviewer #3: I am mostly satisfied with the authors’ answers. There is one subject that needs clarification. LtVTC4 is said to have an SPX domain (lines 31, 95 and 682). However, ref. 61 indicates that TcVTC4 lacks such domain. A quick search in Interpro with the LtVTC4 or the TcVTC4 sequences failed to find an SPX domain. This is important because the SPX domain is the target of inositol pyrophosphates that regulate the VTC activity in yeast and will imply a different regulation mechanism than in yeast.
Please clarify how you detected this domain. If this is not found the statements need to be corrected.

**Response:**

We thank the reviewer for raising this important point.

The assignment of an SPX domain in LtVtc4 was based on structural rather than sequence-based analyses. Recently, Bertolini et al. (53) suggested that trypanosomatid Vtc4 proteins lack an SPX domain based on sequence analysis. Consistent with the reviewer’s observation, InterPro and other sequence-based annotation tools do not identify a canonical SPX domain in LtVtc4 or TcVtc4.

To investigate this further, we performed structural modelling using AlphaFold3. The N-terminal cytoplasmic region of LtVtc4 adopts a well-defined α-helical fold that closely resembles the experimentally solved SPX domain of *Saccharomyces cerevisiae* Vtc4 (PDB ID: 8I6V). A similar fold was also predicted for *Trypanosoma cruzi* Vtc4. Structural superposition demonstrated that the yeast, Leishmania, and Trypanosoma proteins share a conserved overall SPX-domain architecture despite their low sequence conservation (panels A and B in S10 Fig).

Sequence alignment revealed that the trypanosomatid SPX-like regions share only 20.7% sequence identity (LtVtc4-SPX) and 19.5% sequence identity (TcVtc4-SPX) with the yeast ScVtc4-SPX domain (panel C in S10 Fig). This low sequence conservation likely explains why sequence-based domain prediction tools such as InterPro fail to detect the domain.

To clarify this point, we have added S10 Fig showing the structural comparison and sequence alignment of yeast and trypanosomatid Vtc4 SPX domains and revised the manuscript text accordingly (lines 663-684).

PLOS authors have the option to publish the peer review history of their article ([what does this mean?](https://track.editorialmanager.com/CL0/https:%2F%2Fjournals.plos.org%2Fplosntds%2Fs%2Feditorial-and-peer-review-process%23loc-peer-review-history/1/010f019ecf35a0b5-453ca5df-84d9-4339-8d12-11e39054e0eb-000000/xByAEex8KcpT6OQC7XdZtvXT3LpyHw1CfyGA5Q5Fy7U=258)). If published, this will include your full peer review and any attached files.


If you choose “no”, your identity will remain anonymous but your review may still be made public.


**Do you want your identity to be public for this peer review?** For information about this choice, including consent withdrawal, please see our [Privacy Policy](https://track.editorialmanager.com/CL0/https:%2F%2Fwww.plos.org%2Fprivacy-policy/1/010f019ecf35a0b5-453ca5df-84d9-4339-8d12-11e39054e0eb-000000/5SP3yP3SAb5M10bjcJEjksWQ7A1KHFlZyfbQguSOQlI=258).

Reviewer #1: **Yes:** Johannes S. P. Doehl

Reviewer #2: No

Reviewer #3: No

**Figure resubmission:**

While revising your submission, we strongly recommend that you use PLOS’s NAAS tool (<https://ngplosjournals.pagemajik.ai/artanalysis>) to test your figure files. NAAS can convert your figure files to the TIFF file type and meet basic requirements (such as print size, resolution), or provide you with a report on issues that do not meet our requirements and that NAAS cannot fix.
After uploading your figures to PLOS’s NAAS tool - <https://ngplosjournals.pagemajik.ai/artanalysis>, NAAS will process the files provided and display the results in the "Uploaded Files" section of the page as the processing is complete. If the uploaded figures meet our requirements (or NAAS is able to fix the files to meet our requirements), the figure will be marked as "fixed" above. If NAAS is unable to fix the files, a red "failed" label will appear above. When NAAS has confirmed that the figure files meet our requirements, please download the file via the download option, and include these NAAS processed figure files when submitting your revised manuscript.

**Reproducibility:**

To enhance the reproducibility of your results, we recommend that authors of applicable studies deposit laboratory protocols in protocols.io, where a protocol can be assigned its own identifier (DOI) such that it can be cited independently in the future. Additionally, PLOS ONE offers an option to publish peer-reviewed clinical study protocols. Read more information on sharing protocols at <https://plos.org/protocols?utm_medium=editorial-email&utm_source=authorletters&utm_campaign=protocols>
