# Supplementary material for: Novel binding partners of the Vacuolar Transporter Chaperone (VTC) complex in Acidocalcisomes of Leishmania tarentolae
Source: PLoS Negl Trop Dis. 2026 Jul 13;20(7):e0014511. doi: 10.1371/journal.pntd.0014511 (PMC13375126; doi:10.1371/journal.pntd.0014511)
Supplement: Peer review history

# Author comment: Author response to Decision Letter 0

**DOI:** 10.1371/journal.pntd.0014511.r001

**Transfer Alert**

This paper was transferred from another journal. As a result, its full editorial history (including decision letters, peer reviews and author responses) may not be present.

28 Nov 2025

# Peer review: Decision Letter 0

**DOI:** 10.1371/journal.pntd.0014511.r002

**Authors:** Rhoel Ramos Dinglasan, Sarman Singh

18 Jan 2026

PNTD-D-25-02053

Novel Binding Partners of the Vacuolar Transporter Chaperone (VTC) complex in Acidocalcisomes of Leishmania tarentolae

PLOS Neglected Tropical Diseases

Dear Dr. Vidilaseris,

Thank you for submitting your manuscript to PLOS Neglected Tropical Diseases. After careful consideration, we feel that it has merit but does not fully meet PLOS Neglected Tropical Diseases's publication criteria as it currently stands. Therefore, we invite you to submit a revised version of the manuscript that addresses the points raised during the review process.

Please submit your revised manuscript within by Mar 19 2026 11:59PM. If you will need more time than this to complete your revisions, please reply to this message or contact the journal office at plosntds@plos.org. When you're ready to submit your revision, log on to https://www.editorialmanager.com/pntd/ and select the 'Submissions Needing Revision' folder to locate your manuscript file.

Please include the following items when submitting your revised manuscript:

* A letter that responds to each point raised by the editor and reviewer(s). You should upload this letter as a separate file labeled 'Response to Reviewers'. This file does not need to include responses to any formatting updates and technical items listed in the 'Journal Requirements' section below.

* A marked-up copy of your manuscript that highlights changes made to the original version. You should upload this as a separate file labeled 'Revised Manuscript with Track Changes'.

* An unmarked version of your revised paper without tracked changes. You should upload this as a separate file labeled 'Manuscript'.

If you would like to make changes to your financial disclosure, competing interests statement, or data availability statement, please make these updates within the submission form at the time of resubmission. Guidelines for resubmitting your figure files are available below the reviewer comments at the end of this letter.

We look forward to receiving your revised manuscript.

Kind regards,

Rhoel Ramos Dinglasan

Academic Editor

PLOS Neglected Tropical Diseases

Sarman Singh

Section Editor

PLOS Neglected Tropical Diseases

Shaden Kamhawi

co-Editor-in-Chief

PLOS Neglected Tropical Diseases

orcid.org/0000-0003-4304-636XX

Paul Brindley

co-Editor-in-Chief

PLOS Neglected Tropical Diseases

orcid.org/0000-0003-1765-0002

**Additional Editor Comments:**

1- While addressing each reviewer's critique, please pay close attention to a major concern raised by Reviewer 3 with respect to addressing the apparent lack of functional data when revising your manuscript.

2- Please ensure that MIAPE guidelines are followed as there is no data deposition in a recognized international repository. It is important to include gene IDs/Accession numbers for all identified hits in the main text and supplemental data as searchable files.

3- Check through the supplemental data to determine relevance, order of discussion, and appropriateness.

**Journal Requirements:**

If the reviewer comments include a recommendation to cite specific previously published works, please review and evaluate these publications to determine whether they are relevant and should be cited. There is no requirement to cite these works unless the editor has indicated otherwise.

1) We ask that a manuscript source file is provided at Revision. Please upload your manuscript file as a .doc, .docx, .rtf or .tex. If you are providing a .tex file, please upload it under the item type u2018LaTeX Source Fileu2019 and leave your .pdf version as the item type u2018Manuscriptu2019.

2) We do not publish any copyright or trademark symbols that usually accompany proprietary names, eg ©,  ®, or TM  (e.g. next to drug or reagent names). Therefore please remove all instances of trademark/copyright symbols throughout the text, including:

- ® on page: 10

- TM on pages: 8, and 10.

3) Please upload all main figures as separate Figure files in .tif or .eps format. For more information about how to convert and format your figure files please see our guidelines:

https://journals.plos.org/plosntds/s/figures

**Reviewers' Comments:**

Reviewer's Responses to Questions

**Key Review Criteria Required for Acceptance?**

As you describe the new analyses required for acceptance, please consider the following:

**Methods**

-Are the objectives of the study clearly articulated with a clear testable hypothesis stated?

-Is the study design appropriate to address the stated objectives?

-Is the population clearly described and appropriate for the hypothesis being tested?

-Is the sample size sufficient to ensure adequate power to address the hypothesis being tested?

-Were correct statistical analysis used to support conclusions?

-Are there concerns about ethical or regulatory requirements being met?

Reviewer #1: The methods are clearly written and to my understanding sufficiently detailed to be reproducible.

Reviewer #2: The work described in this manuscript was designed to explore the composition of the VTC complex in the Leishmania tarentolae acidocalcisomes. In pursuing this objective, the authors used several methodologies: mass spectrometry, proximity-dependent biotinylation, immunofluorescence microscopy, endogenous labelling of proteins, and structural predictions. As a result, the authors gathered enough information to provide a plausible composition of the VTC complex consisting of two known subunits (VTC1 and VTC4) and three novel VTC binding partners (VBPs). The experimental results are clear and well-illustrated. The structural analyses and the proposed model for the VTC complex are convincing. In sum, this study is a valuable contribution that may serve as a starting point for future ones.

Reviewer #3: The methods used are well described and proper statistical analysis is applied. Information about the source of some reagents (PPase antibodies) is missing.

**********

**Results**

-Does the analysis presented match the analysis plan?

-Are the results clearly and completely presented?

-Are the figures (Tables, Images) of sufficient quality for clarity?

Reviewer #1: The experimental results are well presented and the details sufficient to follow the authors' explanations. However, while the experimental model is Leishmania tarentolae, there are frequent references to T. brucei, which was used to query TryTrip database. It is not always easy to follow, which organism is being discussed, as their is a lot of back and forth, sometimes within a paragraph. Thus, it would be helpful to make sure that it is always clear which organism is being presented at any time in the results section.

The forth paragraphs from the introduction (lines 105 to 111) seem from its content something that would be better placed at the beginning of the results section as it already dives into experimental proceedings. While this is ultimately an editorial decision, I would recommend doing so.

Please, note that supplementary figure 3 is currently being discussed before supplementary figure 2. Please, invert the order of these two figures.

Reviewer #2: See above

Reviewer #3: The results are well presented, and the figures are of good quality. One supplementary figure (Suppl. Fig. 4) appears as taken from a website. Some supplemental figures are not needed.

**********

**Conclusions**

-Are the conclusions supported by the data presented?

-Are the limitations of analysis clearly described?

-Do the authors discuss how these data can be helpful to advance our understanding of the topic under study?

-Is public health relevance addressed?

Reviewer #1: The results provide sufficient evidence to support the authors' conclusion on how the VTC complex is assembled in kinetoplastids, like L. tarentolae, as summarized in figure 9.

Reviewer #2: See above

Reviewer #3: This manuscript reports a study on the components of the vacuolar transporter chaperone (VTC) complex of Leishmania tarentolae. This complex is present in some eukaryotes, has polyphosphate polymerase activity, localizes to vacuoles and acidocalcisomes, and has been well characterized in yeast as possessing 5 subunits (Vtc1-Vtc5). However, only homologs to Vtc1 and the catalytic subunit Vtc4 have been found in trypanosomatids. Using proximity-dependent biotinylation (BioID) the authors found several proteins previously demonstrated to localize to acidocalcisomes in trypanosomatids including 3 putative binding partners, which also localize to acidocalcisomes and interact with LtVtc1 and LtVtc4 in pulldown assays. AlphaFold 3 structural predictions also suggest their interaction with the complex.

**********

**Editorial and Data Presentation Modifications?**

Use this section for editorial suggestions as well as relatively minor modifications of existing data that would enhance clarity. If the only modifications needed are minor and/or editorial, you may wish to recommend “Minor Revision” or “Accept”.

Reviewer #1: Overall, this is very well structure manuscript. With the exception of the couple of comments in the results section, I have no further comments here.

Reviewer #2: Although the manuscript is well-written, several issues require modification or clarification.

Specific points.

1. Line 142. Pen-Strep likely refers to an antibiotic mixture, but this should be clarified. Also, it should be indicated whether this mixture was used as a common component of the growth media or used specifically at this stage.

2. Line 198. The generation of the anti-mPPase antibody should be briefly commented on.

3. Line 223-224: The sentence "the peptide... recovered" has a cryptic meaning. Please, clarify.

4. Line 245. Please, specify what IP6 is.

5. If DDM is not a standard abbreviation and may have different meanings, please indicate (first time it is mentioned) that it corresponds to dodecyl-beta-maltoside (as it is indicated in the supplementary fig 1 legend). This compound is extensively used in this work and merits avoiding any confusion about its nature. The same apply for FC-12 (Fos-choline-12).

6. Line 261. Please, specify what "fluorescent protein marker" was used

7. Line 310. To facilitate the identification of orthologs for LtVtc1 and LtVtc4 in other kinetoplastids, the inclusion (here or in a previous section) of the TriTryDB gene IDs for these Lt genes would be valuable.

8. Lines 343-344. It is unclear what “1240 out of the 3160 identified proteins are unique hits” means. Could you rephrase the sentence for clarity?

Moreover, an Excel file with the proteins identified in each one of the four datasets would be a valuable resource. Therefore, this reviewer encourages authors to provide this information within a supplementary file.

9. Line 365. How are poly-P binding proteins identified? Is there any structural feature that was used to make the selection? Please, clarify this.

10. Lines 609-610. The article quoted as reference 60 deals with a T. cruzi WD-repeat containing protein. Please, check if referring to L. major in this sentence is appropriate.

11. The authors should consider depositing the proteomics raw data in a repository like PRIDE or similar.

Reviewer #3: Major points: The work provides confirmation for the acidocalcisome localization of several proteins and describes new putative components of the VTC complex in Leishmania. However, there is no evidence presented on the enzymatic activity of the complex, on how this is affected by the three putative binding partners, or on the essentiality of the new components for the life cycle of L. tarentolae. Experimental evidence on these aspects, as well as some corrections, needs to be provided in order to substantiate the conclusions and improve the quality of this work.

Minor points:

1. Fig. 1B shows bands of the same size for mNG-VTC1 and mNG-VTC4. Is this right? Fig. 1D shows co-localization with mPPase antibodies. Which is the source of these antibodies? Is this tagged PPase?

2. Fig. 2. An Excel file with the list of proteins identified in different experiments will be appropriate as supplemental information.

3. Table 1. There are no Vacuolar-P-type ATPases. The name vacuolar should be used only for the V-ATPases. V-ATPases are different from P-type ATPases.

4. Suppl. Fig. 1A shows bands of the same size for BirA+-VTC1 and BirA+-VTC4. Is this right? Fig. 1H is not explained. Loading controls are missing in the western blots and should be provided. Which is the source of TmPPase antibody?

5. Suppl. Fig. 2 is not mentioned in the text.

6. Suppl. Fig. 4. Is this taken from a website? Do you have permission to reproduce it?

7. Suppl. Figs. 7 and 10. These figures are not necessary.

8. Suppl. Tables 2-5 can be replaced with an Excel file listing all the proteins identified without attributing localization, or polyphosphorylation, as the attribution indicates only a possibility that for the most part has not been validated.

**********

**Summary and General Comments**

Use this section to provide overall comments, discuss strengths/weaknesses of the study, novelty, significance, general execution and scholarship. You may also include additional comments for the author, including concerns about dual publication, research ethics, or publication ethics. If requesting major revision, please articulate the new experiments that are needed.

Reviewer #1: The manuscript titled "Novel Binding Partners of the Vacuolar Transporter Chaperone (VTC) complex in Acidocalcisomes of Leishmania tarentolae" is technically very dense work. It provides sufficient details to insure that experimental execution was successful and that presented results hold up to interpretation. The only weakness I am currently seeing is that the manuscript it not always easy to follow and may be a challenge for an interested reader from the Leishmania field, who is not that deep into proteomics. While I understand that it is challenging in a technically dense study to be generally comprehensible, I believe it would benefit the study to be clearer on when the authors refer to L. tarentolae and when to T. brucei. Make sure that it is clear, when you target the whole acidocalcisome and when only the protein(s) in an assay. Make it clearer to the reader what the GO term analysis is telling us. It may also be worth to include in the discussion what the authors' findings mean to the field of kinetoplastid research; why does it help the field to know the components of the VTC complex?

Beyond a bit more clarity, I do not see any needs for more experiments.

Reviewer #2: See above

Reviewer #3: The manuscript presents results showing proximity-dependent biotinylation (BioID) assays of VTC subunits that identify 3 putative binding partners, which localize to acidocalcisomes and interact with LtVtc1 and LtVtc4 in pulldown assays. AlphaFold 3 structural predictions also suggest their interaction with the complex. However, there is no evidence of the polyphosphate polymerase activity of the complex or that there is a complex and of the role of the binding partners in the activity of the complex.

The results are interesting. However, evidence on the enzymatic activity of the complex, on the role of putative binding partners in the formation of a complex, and on their effect on its activity will greatly enhance the value of the paper.

**********

PLOS authors have the option to publish the peer review history of their article (what does this mean?). If published, this will include your full peer review and any attached files.

If you choose “no”, your identity will remain anonymous but your review may still be made public.

**Do you want your identity to be public for this peer review?**   For information about this choice, including consent withdrawal, please see our Privacy Policy.

Reviewer #1: No

Reviewer #2: No

Reviewer #3: No

**Figure resubmission:**

While revising your submission, we strongly recommend that you use PLOS’s NAAS tool (https://ngplosjournals.pagemajik.ai/artanalysis) to test your figure files. NAAS can convert your figure files to the TIFF file type and meet basic requirements (such as print size, resolution), or provide you with a report on issues that do not meet our requirements and that NAAS cannot fix.

After uploading your figures to PLOS’s NAAS tool - https://ngplosjournals.pagemajik.ai/artanalysis, NAAS will process the files provided and display the results in the "Uploaded Files" section of the page as the processing is complete. If the uploaded figures meet our requirements (or NAAS is able to fix the files to meet our requirements), the figure will be marked as "fixed" above. If NAAS is unable to fix the files, a red "failed" label will appear above. When NAAS has confirmed that the figure files meet our requirements, please download the file via the download option, and include these NAAS processed figure files when submitting your revised manuscript.

**Reproducibility:**

To enhance the reproducibility of your results, we recommend that authors of applicable studies deposit laboratory protocols in protocols.io, where a protocol can be assigned its own identifier (DOI) such that it can be cited independently in the future. Additionally, PLOS ONE offers an option to publish peer-reviewed clinical study protocols. Read more information on sharing protocols at https://plos.org/protocols?utm_medium=editorial-email&utm_source=authorletters&utm_campaign=protocols

# Author comment: Author response to Decision Letter 1

**DOI:** 10.1371/journal.pntd.0014511.r003

21 May 2026

Attachment Submitted filename: Response to Reviewers_revision2.docx

# Peer review: Decision Letter 1

**DOI:** 10.1371/journal.pntd.0014511.r004

**Authors:** Rhoel Ramos Dinglasan, Sarman Singh

16 Jun 2026

PNTD-D-25-02053R1Novel Binding Partners of the Vacuolar Transporter Chaperone (VTC) complex in Acidocalcisomes of Leishmania tarentolaePLOS Neglected Tropical Diseases Dear Dr. Vidilaseris, Thank you for submitting your manuscript to PLOS Neglected Tropical Diseases. After careful consideration, we feel that it has merit but does not fully meet PLOS Neglected Tropical Diseases's publication criteria as it currently stands. Therefore, we invite you to submit a revised version of the manuscript that addresses the points raised during the review process. Please submit your revised manuscript by Aug 15 2026 11:59PM. If you will need more time than this to complete your revisions, please reply to this message or contact the journal office at plosntds@plos.org. When you're ready to submit your revision, log on to https://www.editorialmanager.com/pntd/ and select the 'Submissions Needing Revision' folder to locate your manuscript file. Please include the following items when submitting your revised manuscript:* A letter that responds to each point raised by the editor and reviewer(s). You should upload this letter as a separate file labeled 'Response to Reviewers'. This file does not need to include responses to any formatting updates and technical items listed in the 'Journal Requirements' section below.* A marked-up copy of your manuscript that highlights changes made to the original version. You should upload this as a separate file labeled 'Revised Manuscript with Track Changes'.* An unmarked version of your revised paper without tracked changes. You should upload this as a separate file labeled 'Manuscript'. If you would like to make changes to your financial disclosure, competing interests statement, or data availability statement, please make these updates within the submission form at the time of resubmission. Guidelines for resubmitting your figure files are available below the reviewer comments at the end of this letter. As the corresponding author, your ORCID iD is verified in the submission system and will appear in the published article. PLOS supports the use of ORCID, and we encourage all coauthors to register for an ORCID iD and use it as well. Please encourage your coauthors to verify their ORCID iD within the submission system before final acceptance, as unverified ORCID iDs will not appear in the published article. Only the individual author can complete the verification step; PLOS staff cannot verify ORCID iDs on behalf of authors.We look forward to receiving your revised manuscript. Kind regards, Rhoel Ramos DinglasanAcademic EditorPLOS Neglected Tropical Diseases Sarman SinghSection EditorPLOS Neglected Tropical Disease

Shaden Kamhawi

co-Editor-in-Chief

PLOS Neglected Tropical Diseases

orcid.org/0000-0003-4304-636XX

Paul Brindley

co-Editor-in-Chief

PLOS Neglected Tropical Diseases

orcid.org/0000-0003-1765-0002

**Reviewers' comments:**   Reviewer's Responses to Questions

**Key Review Criteria Required for Acceptance?**

As you describe the new analyses required for acceptance, please consider the following:

**Methods**

-Are the objectives of the study clearly articulated with a clear testable hypothesis stated?

-Is the study design appropriate to address the stated objectives?

-Is the population clearly described and appropriate for the hypothesis being tested?

-Is the sample size sufficient to ensure adequate power to address the hypothesis being tested?

-Were correct statistical analysis used to support conclusions?

-Are there concerns about ethical or regulatory requirements being met?

Reviewer #1: No further comments

Reviewer #2: As stated in my previous report, this study is well-designed and well-executed. The objectives were clearly articulated. Correct statistical analyses were used.

Reviewer #3: Methods are acceptable

**********

**Results**

-Does the analysis presented match the analysis plan?

-Are the results clearly and completely presented?

-Are the figures (Tables, Images) of sufficient quality for clarity?

Reviewer #1: No further comments

Reviewer #2: The results are clear and exhaustive. The figures, tables, and supplementary materials are of sufficient quality.

Reviewer #3: Authors addressed most of the concerns

**********

**Conclusions**

-Are the conclusions supported by the data presented?

-Are the limitations of analysis clearly described?

-Do the authors discuss how these data can be helpful to advance our understanding of the topic under study?

-Is public health relevance addressed?

Reviewer #1: No further comments

Reviewer #2: The conclusions are based on solid experimental data, and limitations are clearly described. Also, the authors comment on the usefulness of this study for future work.

Reviewer #3: Yes, conclusions support the data

**********

**Editorial and Data Presentation Modifications?**

Use this section for editorial suggestions as well as relatively minor modifications of existing data that would enhance clarity. If the only modifications needed are minor and/or editorial, you may wish to recommend “Minor Revision” or “Accept”.

Reviewer #1: No further comments

Reviewer #2: I agree with the way the authors have addressed my previous suggestions. Moreover, new experiments were added that increase the relevance of this work. No modifications are needed.

Reviewer #3: See comments under the Summary section

**********

**Summary and General Comments**

Use this section to provide overall comments, discuss strengths/weaknesses of the study, novelty, significance, general execution and scholarship. You may also include additional comments for the author, including concerns about dual publication, research ethics, or publication ethics. If requesting major revision, please articulate the new experiments that are needed.

Reviewer #1: Accept

Reviewer #2: This study is a valuable contribution to our understanding of the composition of the VTC complex in the Leishmania tarentolae acidocalcisomes. Moreover, it contains structural predictions that may guide future studies. The methodologies described may be of value for other researchers.

Reviewer #3: I am mostly satisfied with the authors’ answers. There is one subject that needs clarification. LtVTC4 is said to have an SPX domain (lines 31, 95 and 682). However, ref. 61 indicates that TcVTC4 lacks such domain. A quick search in Interpro with the LtVTC4 or the TcVTC4 sequences failed to find an SPX domain. This is important because the SPX domain is the target of inositol pyrophosphates that regulate the VTC activity in yeast and will imply a different regulation mechanism than in yeast.

Please clarify how you detected this domain. If this is not found the statements need to be corrected.

**********

PLOS authors have the option to publish the peer review history of their article (what does this mean?). If published, this will include your full peer review and any attached files.

If you choose “no”, your identity will remain anonymous but your review may still be made public.

**Do you want your identity to be public for this peer review?**   For information about this choice, including consent withdrawal, please see our Privacy Policy.

Reviewer #1: **Yes:**  Johannes S. P. Doehl

Reviewer #2: No

Reviewer #3: No

[NOTE: If reviewer comments were submitted as an attachment file, they will be attached to this email and accessible via the submission site. Please log into your account, locate the manuscript record, and check for the action link "View Attachments". If this link does not appear, there are no attachment files.] **Figure resubmission:**    While revising your submission, we strongly recommend that you use PLOS’s NAAS tool (https://ngplosjournals.pagemajik.ai/artanalysis) to test your figure files. NAAS can convert your figure files to the TIFF file type and meet basic requirements (such as print size, resolution), or provide you with a report on issues that do not meet our requirements and that NAAS cannot fix.

After uploading your figures to PLOS’s NAAS tool - https://ngplosjournals.pagemajik.ai/artanalysis, NAAS will process the files provided and display the results in the "Uploaded Files" section of the page as the processing is complete. If the uploaded figures meet our requirements (or NAAS is able to fix the files to meet our requirements), the figure will be marked as "fixed" above. If NAAS is unable to fix the files, a red "failed" label will appear above. When NAAS has confirmed that the figure files meet our requirements, please download the file via the download option, and include these NAAS processed figure files when submitting your revised manuscript. **Reproducibility:**   To enhance the reproducibility of your results, we recommend that authors of applicable studies deposit laboratory protocols in protocols.io, where a protocol can be assigned its own identifier (DOI) such that it can be cited independently in the future. Additionally, PLOS ONE offers an option to publish peer-reviewed clinical study protocols. Read more information on sharing protocols at https://plos.org/protocols?utm_medium=editorial-email&utm_source=authorletters&utm_campaign=protocols

# Author comment: Author response to Decision Letter 2

**DOI:** 10.1371/journal.pntd.0014511.r005

25 Jun 2026

Attachment Submitted filename: Response_to_Reviewers_Revision2_auresp_2.docx

# Editor report: Decision Letter 2

**DOI:** 10.1371/journal.pntd.0014511.r006

**Authors:** Rhoel Ramos Dinglasan, Sarman Singh

26 Jun 2026

Dear Vidilaseris,

We are pleased to inform you that your manuscript 'Novel Binding Partners of the Vacuolar Transporter Chaperone (VTC) complex in Acidocalcisomes of Leishmania tarentolae' has been provisionally accepted for publication in PLOS Neglected Tropical Diseases.

Before your manuscript can be formally accepted you will need to complete some formatting changes, which you will receive in a follow up email. A member of our team will be in touch with a set of requests.

Please note that your manuscript will not be scheduled for publication until you have made the required changes, so a swift response is appreciated.

IMPORTANT: The editorial review process is now complete. PLOS will only permit corrections to spelling, formatting or significant scientific errors from this point onwards. Requests for major changes, or any which affect the scientific understanding of your work, will cause delays to the publication date of your manuscript.

Should you, your institution's press office or the journal office choose to press release your paper, you will automatically be opted out of early publication. We ask that you notify us now if you or your institution is planning to press release the article. All press must be co-ordinated with PLOS.

Thank you again for supporting Open Access publishing; we are looking forward to publishing your work in PLOS Neglected Tropical Diseases.

Best regards,

Rhoel Ramos Dinglasan

Academic Editor

PLOS Neglected Tropical Diseases

Sarman Singh

Section Editor

PLOS Neglected Tropical Diseases

Shaden Kamhawi

co-Editor-in-Chief

PLOS Neglected Tropical Diseases

orcid.org/0000-0003-4304-636XX

Paul Brindley

co-Editor-in-Chief

PLOS Neglected Tropical Diseases

orcid.org/0000-0003-1765-0002

***********************************************************

# Editor report: Acceptance letter

**DOI:** 10.1371/journal.pntd.0014511.r007

**Authors:** Rhoel Ramos Dinglasan, Sarman Singh

Dear Vidilaseris,

We are delighted to inform you that your manuscript, "Novel Binding Partners of the Vacuolar Transporter Chaperone (VTC) complex in Acidocalcisomes of Leishmania tarentolae," has been formally accepted for publication in PLOS Neglected Tropical Diseases.

We have now passed your article onto the PLOS Production Department who will complete the rest of the publication process. All authors will receive a confirmation email upon publication.

The corresponding author will soon be receiving a typeset proof for review, to ensure errors have not been introduced during production. Please review the PDF proof of your manuscript carefully, as this is the last chance to correct any scientific or type-setting errors. Please note that major changes, or those which affect the scientific understanding of the work, will likely cause delays to the publication date of your manuscript. Note: Proofs for Front Matter articles (Editorial, Viewpoint, Symposium, Review, etc...) are generated on a different schedule and may not be made available as quickly.

Soon after your final files are uploaded, the early version of your manuscript will be published online unless you opted out of this process. The date of the early version will be your article's publication date. The final article will be published to the same URL, and all versions of the paper will be accessible to readers.

For Research Articles, you will receive an invoice from PLOS for your publication fee after your manuscript has reached the completed accept phase. If you receive an email requesting payment before acceptance or for any other service, this may be a phishing scheme. Learn how to identify phishing emails and protect your accounts at https://explore.plos.org/phishing.

Thank you again for supporting open-access publishing; we are looking forward to publishing your work in PLOS Neglected Tropical Diseases.

Best regards,

Shaden Kamhawi

co-Editor-in-Chief

PLOS Neglected Tropical Diseases

Paul Brindley

co-Editor-in-Chief

PLOS Neglected Tropical Diseases
